# Integrated Proteomic and Metabolomic Profiling for Developing Novel Plasma‐Based Diagnostic Models of Sarcopenia

**DOI:** 10.1002/jcsm.70188

**Published:** 2026-01-16

**Authors:** Dongqin Xu, Haoran Jin, Jinlin Yang, Zhiliang Zuo, Rui Ou, Fengjuan Hu, Lu Pu, Yuxing Dong, Meng Wu, Birong Dong, Hao Jiang

**Affiliations:** ^1^ Laboratory for Aging and Cancer Research, National Clinical Research Center for Geriatrics and State Key Laboratory of Respiratory Health and Multimorbidity, West China Hospital Sichuan University Chengdu China; ^2^ Laboratory of Aging and Cancer, Frontiers Science Center for Disease‐Related Molecular Network, West China Hospital Sichuan University Chengdu China; ^3^ Department of Gastroenterology and Hepatology, West China Hospital Sichuan University Chengdu China; ^4^ Sichuan University‐University of Oxford Huaxi Joint Centre for Gastrointestinal Cancer, Frontiers Science Center for Disease‐Related Molecular Network, West China Hospital Sichuan University Chengdu China; ^5^ Center of Gerontology and Geriatrics, National Clinical Research Center for Geriatrics, West China Hospital Sichuan University Chengdu Sichuan China; ^6^ School of Sports Medicine and Health Chengdu Sport University Chengdu China; ^7^ PICC Health Insurance Company Limited Beijing China

**Keywords:** combined model, diagnosis, machine learning, metabolomic, proteomic, sarcopenia

## Abstract

**Background:**

Sarcopenia is a progressive, age‐related condition characterized by a decline in skeletal muscle mass, strength and performance. Diagnosis remains challenging because current consensus criteria are difficult to scale and existing biomarkers lack accuracy. This study aimed to develop high‐performance plasma‐based diagnostic models for sarcopenia by integrating proteomic and metabolomic profiles.

**Methods:**

Participants were selected from the West China Health and Aging Trend study. Sarcopenia was defined according to the 2019 Asian Working Group for Sarcopenia (AWGS) criteria. Two independent 1:1 age‐ and sex‐matched cohorts were constructed: a discovery cohort (40 sarcopenic, 40 non‐sarcopenic) and a validation cohort (30 sarcopenic, 30 non‐sarcopenic). Fasting plasma samples were profiled using the Olink Explore 384 Inflammation Panel and liquid chromatography–mass spectrometry‐based untargeted metabolomics. Gaussian naïve Bayes classifiers were trained for single‐omics models, and logistic regression was used to construct combined models in the discovery cohort and evaluate performance in the validation cohort.

**Results:**

Baseline age and sex were similar in sarcopenic and non‐sarcopenic groups (discovery: median 72.0 vs. 71.5 years, *p* = 0.714; validation: 71.0 vs. 71.5 years, *p* = 0.594; women: 52.5% and 53.3%). The sarcopenic group had lower skeletal muscle index, grip strength and gait speed (all *p* < 0.05). Sixty‐five proteins and 268 metabolites differed between groups. A 7‐protein Gaussian naïve Bayes model achieved AUCs of 0.743 (95% CI 0.718–0.767) in discovery and 0.698 (0.561–0.834) in validation; the metabolomic model yielded 0.828 (0.808–0.849) and 0.751 (0.617–0.885). Combined Model 1 integrated the probabilistic outputs of the proteomic (7 proteins) and metabolomic (7 metabolites) models and reached AUCs of 0.951 (0.937–0.965) and 0.823 (0.717–0.930), outperforming single‐omics models (discovery: both *p* < 0.001; validation: vs. proteomic *p* < 0.05; vs. metabolomic *p* = 0.147). Combined Model 2 incorporated only the top two biomarkers from each platform (CCL13, FGF2, N‐hexadecanoylpyrrolidine and 1‐(cyclohexylmethyl)proline), achieving AUCs of 0.853 (0.828–0.878) in discovery and 0.911 (0.839–0.983) in validation and remained superior to single‐omics models (discovery: both *p* < 0.001; validation: both *p* < 0.05). Its validation performance was comparable to Combined Model 1 (*p* = 0.124), with sensitivity 86.7%, specificity 80.0%, precision 81.2% and F1‐score 0.839.

**Conclusions:**

We have developed high‐performance plasma‐based diagnostic models for sarcopenia by integrating inflammatory proteomic and metabolomic signatures. A four‐biomarker model (Combined Model 2) demonstrated excellent diagnostic performance and may provide a promising clinically scalable approach for the early detection of sarcopenia.

## 1 Introduction

Sarcopenia is a progressive skeletal muscle disorder characterized by the loss of muscle mass, strength and physical performance. This age‐related disease is a critical public health concern in aged populations worldwide. Epidemiological studies have revealed that approximately 10%–20% of individuals aged 60 years or older are affected, with prevalence rates increasing to over 30% in those aged 80 years or above [1]. In addition to severely diminishing a sufferer's quality of life, sarcopenia also imposes a substantial healthcare burden, driven by heightened risks of falls, fractures and hospitalization [2]. Despite the significant clinical and socioeconomic impact of sarcopenia, no pharmacological therapies are currently approved by any regulatory agency, and current management strategies, primarily resistance exercise and nutritional supplementation, exhibit limited efficacy in advanced‐stage patients [3]. Early diagnosis is therefore pivotal to delaying disease progression and improving outcomes, yet existing diagnostic frameworks face significant practical challenges.

The European Working Group on Sarcopenia in Older People (EWGSOP2), International Classification of Disease (ICD‐11) and Asian Working Group for Sarcopenia (AWGS) criteria remain the gold standards for diagnosis, and these rely on assessments of muscle mass, grip strength and gait speed. However, these methods are hindered by their reliance on specialized equipment (dual‐energy X‐ray absorptiometry), time‐consuming protocols and poor scalability for population‐wide screening [4]. Consequently, plasma biomarkers have garnered significant attention for use as streamlined diagnostic tools, especially given their ability to reflect the multifactorial pathophysiology of sarcopenia—which includes chronic inflammation, mitochondrial dysfunction, oxidative stress and metabolic dysregulation [5, 6]. While studies have identified several biomarker candidates—for example, the creatinine‐to‐cystatin C ratio, the aspartate aminotransferase‐to‐alanine aminotransferase ratio and cell‐free mitochondrial DNA—their diagnostic performance in validation cohorts remains suboptimal, with sensitivity and specificity rarely exceeding 80% [7]. These outcomes underscore an urgent need to discover novel biomarker signatures and diagnostic panels.

Recent advancements in high‐throughput omics technologies offer unprecedented opportunities to unravel complex disease mechanisms and identify robust biomarkers. Plasma proteomics, particularly via the Olink platform, has revolutionized protein quantification by employing proximity extension assay (PEA) technology to measure low‐abundance proteins at attomolar sensitivity using minimal sample volumes [8]. This approach overcomes the limitations of traditional methods such as enzyme‐linked immunosorbent assay (ELISA) and mass spectrometry, which often lack the dynamic range or reproducibility required for large‐scale studies. Additionally, untargeted metabolomics leveraging liquid chromatography–mass spectrometry (LC–MS) has enabled the unbiased detection of thousands of metabolites, providing insights into dysregulated pathways such as amino acid catabolism, lipid oxidation and energy metabolism [9]. An integration of these proteomics and metabolomics methods may facilitate the elucidation of disease mechanisms and facilitate the discovery of combinatorial biomarker panels with enhanced diagnostic accuracy. Further support for the synergistic potential of multi‐omics integration is continuously emerging. For instance, combined proteomic‐metabolomic analyses have identified novel signatures for neurodegenerative and cardiovascular diseases, demonstrating superior diagnostic performance compared to single‐omics approaches [10, 11]. In sarcopenia, such strategies remain underexplored for diagnostic purposes, despite the growing recognition of its heterogeneous aetiology.

In the present study, we address these gaps by integrating Olink proteomics (Explore 384 Inflammation Panel) and LC–MS‐based untargeted metabolomics techniques to profile plasma samples from sarcopenia cohorts. We aim to (1) identify dysregulated proteins and metabolites associated with sarcopenia, (2) construct diagnostic models with optimized sensitivity and specificity by utilizing a machine learning‐driven framework and (3) validate these biomarkers in independent populations. Together, these findings should enhance current understanding of the molecular underpinnings of sarcopenia and establish a foundation for the development of scalable, non‐invasive diagnostic tools.

## 2 Materials and Methods

### 2.1 Participants

This study has been registered with the Chinese Clinical Trial Registry (ChiCTR1800018895). The study population was derived from the West China Health and Aging Trend (WCHAT) study, an ongoing community‐based cohort initiated in 2018 that enrolled middle‐aged and older adults from Western China. All participants provided written informed consent. Ethical approval was granted by the Institutional Ethics Committee of West China Hospital, Sichuan University, and the collection of biological specimens was approved by the Human Genetic Resources Administration of China ([2019]791).

### 2.2 Study Design

**FIGURE 1:**
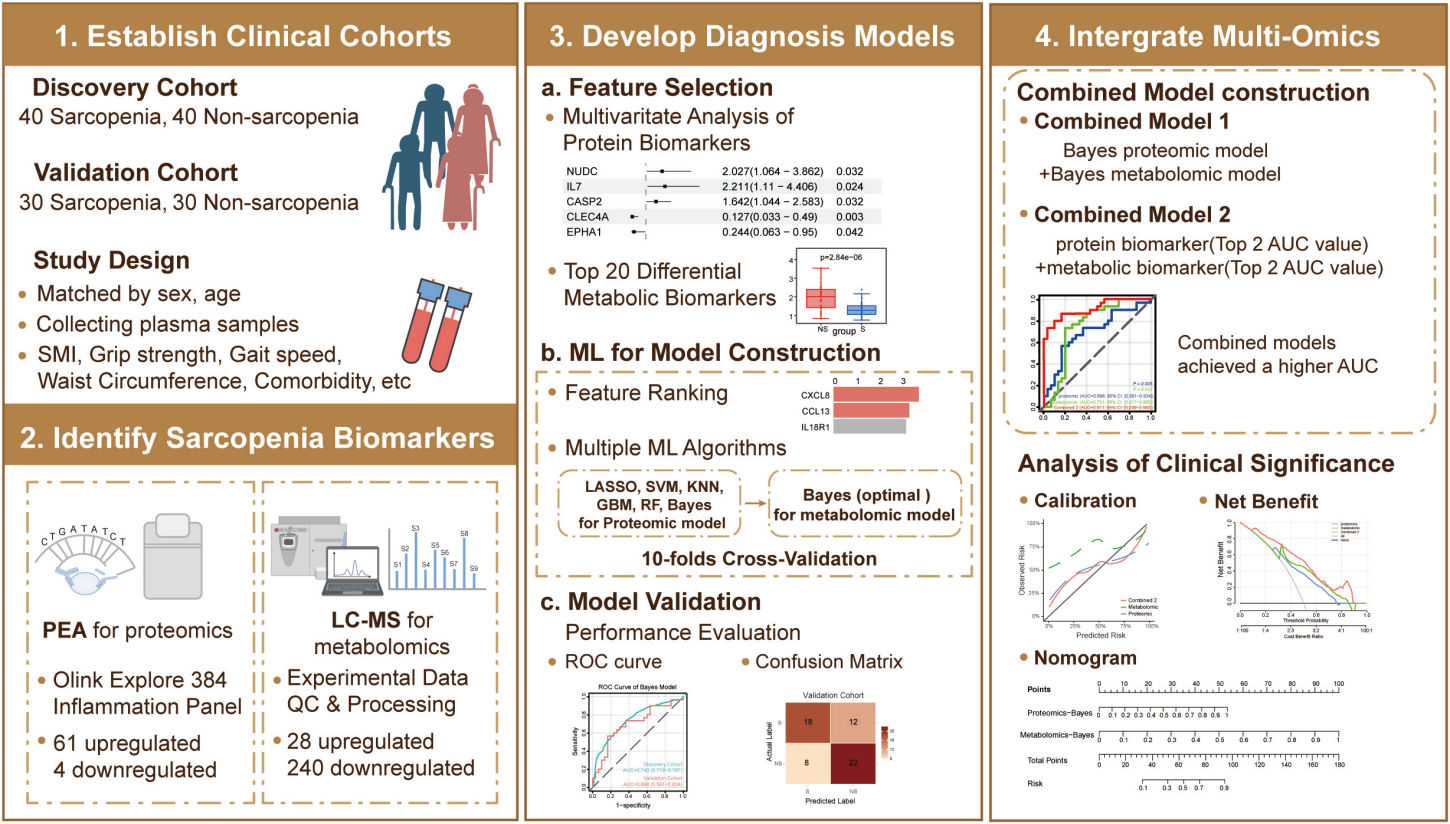
Schematic workflow of this research. BMI, body mass index; LC–MS, liquid chromatography–mass spectrometry; ML, machine learning; PEA, proximity extension assay; SMI, skeletal muscle index.

**FIGURE 2:**
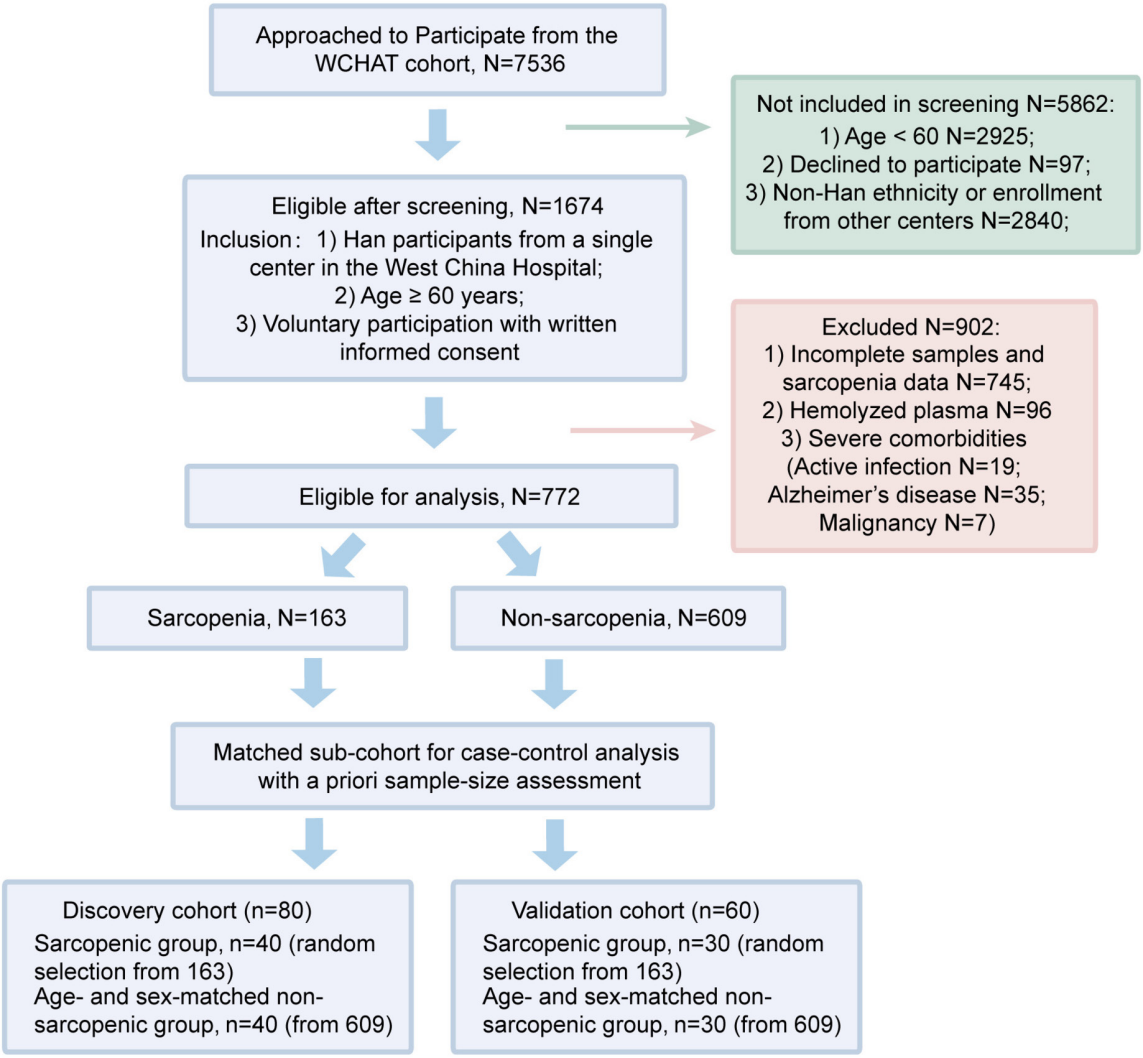
CONSORT flowchart of participant recruitment.

A retrospective, case–control study was conducted within the WCHAT cohort to evaluate the utility of plasma biomarkers for the diagnosis of sarcopenia (overall schema in Figure 1). The WCHAT study originally recruited 7536 community residents [12, 13]. For the present analysis, the inclusion criteria were as follows: (1) Han Chinese participants who attended the WCHAT baseline survey at West China Hospital; (2) age ≥ 60 years; and (3) voluntary participation with written informed consent. Exclusion criteria were as follows: (1) incomplete sarcopenia assessments; (2) hemolyzed plasma samples; and (3) severe comorbidities likely to substantially affect muscle status or biomarker profiles, including active infection, Alzheimer's disease and malignancy. Non‐sarcopenic participants were then selected according to a case–control design. The recruitment flowchart (Figure 2) details the numbers screened, reasons for exclusion and loss and final enrollment. Participants were subsequently allocated to discovery and validation cohorts. Next, diagnostic proteins and metabolites were identified using the proximity extension assay (PEA) and liquid chromatography–mass spectrometry (LC–MS), respectively. Identified biomarkers were used to construct diagnostic models (a proteomic model, a metabolomic model and two combined models) from the optimal machine learning algorithm. These models were then evaluated in an independent validation cohort to assess diagnostic performance. Finally, combined models were further evaluated for predictive performance and potential clinical utility.

### 2.3 Diagnosis of Sarcopenia

Sarcopenia was defined according to the 2019 criteria of the Asian Working Group for Sarcopenia (AWGS). The diagnosis required the presence of low muscle mass, low muscle function, and poor physical performance. Skeletal muscle mass index (SMI) was measured by bioelectrical impedance analysis (BIA; InBody 770, InBody Co., Seoul, South Korea). BIA was performed under standardized conditions: participants fasted for at least 8 h, avoided vigorous exercise and alcohol for 24 h, emptied their bladder on arrival and then stood barefoot on the analyser platform in light clothing with all metal accessories removed, holding the hand electrodes according to the manufacturer's instructions; individuals with cardiac pacemakers or major metallic implants were not evaluated by BIA and were excluded from SMI‐based classification. Low muscle mass was defined as SMI < 7.0 kg/m^2^ in men and < 5.7 kg/m^2^ in women. Handgrip strength was measured with an electronic dynamometer (EH101; Camry, Zhongshan, China) with participants standing upright (or seated if unable to stand), feet shoulder‐width apart and the tested arm naturally extended alongside the body without contact with other body parts; after one familiarization attempt, two maximal efforts were performed for each hand with at least 30 s of rest, and the highest value from either hand was used. Low muscle strength was defined as < 28 kg in men and < 18 kg in women. Gait speed was assessed over a 6‐m walk at the participant's normal pace while wearing their usual footwear; walking aids were allowed if regularly used but no physical assistance was given. Time to traverse the central 6 m was recorded, two trials were performed, gait speed (m/s) was calculated as distance divided by time and the faster value was used. Poor physical performance was defined as gait speed < 1.0 m/s, in line with AWGS 2019 [14].

### 2.4 Sample‐Size Assessment

We conducted an a priori sample‐size assessment. Using the Hanley–McNeil approach (H_0_: AUC = 0.5; two‐sided α = 0.05; power = 80%; anticipated AUC = 0.80; 1:1 case–control ratio), the validation set required approximately 60 participants. For precision, DeLong's variance approximation indicated a 95% confidence‐interval half‐width of ~±0.11 with 30 cases and 30 controls at AUC = 0.80. Accordingly, we planned and achieved a 40/40 discovery cohort for model development with internal validation and a 30/30 validation cohort for external evaluation, meeting both power and prespecified precision targets.

### 2.5 Sample and Clinical Data Collection

Morning fasting peripheral venous blood was collected in EDTA (lavender‐top) tubes in the morning to reduce dietary and circadian variability. Plasma samples were obtained by centrifugation at 3000 rpm for 10 min, and these were timely aliquoted and stored at −80°C. Baseline clinical data included demographic information (age, sex and BMI), sarcopenia‐related physical measures (SMI, grip strength, gait speed, calf circumference, mid‐upper arm circumference, waist circumference, hip circumference and triceps skinfold thickness) and age‐related comorbidities (hypertension, coronary heart disease, stroke and diabetes).

### 2.6 Proteomics Detection

The Olink Explore 384 Inflammation panel was selected to identify sarcopenia‐related protein biomarkers from an inflammatory perspective. For the measurements, 369 pairs of antibodies, each conjugated to a unique single‐stranded DNA oligonucleotide (‘probe DNA’), were added to 1 μL of plasma samples. When both antibodies were bound to the same target protein, the attached DNA probes were brought into close proximity, allowing them to hybridize via complementary sequences and form a double‐stranded DNA template. This template is extended by DNA polymerase and subsequently amplified using next‐generation sequencing. In this microliter‐scale reaction, the amount of amplified DNA is positively correlated with the concentration of the target protein. Proteomic data were processed using the Olink standard NPX pipeline implemented in Olink Insight, which minimizes batch/plate effects through (i) within‐plate normalization using extension controls, (ii) inter‐plate control (IPC) normalization across plates and (iii) intensity normalization to the log2 NPX scale. Manufacturer quality control (QC) metrics were applied before downstream analysis. All the above analytical procedures were performed by Shanghai Applied Protein Technology Co. Ltd., Shanghai, China (https://www.aptbiotech.com/).

### 2.7 Untargeted Metabolomics Analysis

LC–MS‐based untargeted metabolomics was performed by Shanghai Biotree Biotech Co. Ltd. First, metabolites were extracted from 50 μL of plasma using a biphasic solvent system consisting of 200 μL of pre‐chilled methanol: acetonitrile (1:1, v/v) containing 0.1% formic acid. The mixture was then vortexed, subjected to ultrasonication at 4°C and then centrifuged. The resulting supernatant was vacuum‐dried and reconstituted in 100 μL of acetonitrile:water (1:1, v/v) containing 10 ppm of the deuterated internal standard L‐2‐chlorophenylalanine.

Chromatographic separation was performed using a Waters ACQUITY UPLC BEH Amide column under a gradient elution program with water (mobile phase A) and acetonitrile (mobile phase B) at a flow rate of 0.3 mL/min. High‐resolution mass spectrometric analysis was conducted on an Orbitrap Exploris 120 system operated in both positive and negative ionization modes (*m*/*z*: 70–1050). Optimized parameters were as follows: sheath gas flow rate, 50 Arb; auxiliary gas flow rate, 15 Arb; capillary temperature, 320°C; spray voltage, 3.0 kV. Raw data were converted into mzXML format using ProteoWizard software. Metabolite identification was conducted using a custom‐developed R package in conjunction with the BiotreeDB (V3.0) database.

### 2.8 Statistical and Bioinformatics Analysis

Statistical analyses were performed in SPSS 25.0. Continuous variables were assessed for normality using the Shapiro–Wilk test (α = 0.05). Normally distributed data are reported as mean ± SD and compared with Student's *t*‐test; non‐normal data are reported as median (IQR) and compared with the Wilcoxon rank‐sum (Mann–Whitney *U*) test. Categorical variables were compared using the chi‐square test or Fisher's exact test, as appropriate. All tests were two‐sided with α = 0.05.

Principal component analysis (PCA) was used to evaluate the degree of separation between two groups. Protein levels between sarcopenia and non‐sarcopenia were compared using either a Student's *t*‐test or a Wilcoxon rank‐sum test according to distributional assumptions. Differential expression patterns were visualized using a volcano plot (*p*‐values vs. log_2_ fold changes) and a hierarchical clustering heatmap. Functional annotation of identified proteins was performed using DAVID for Gene Ontology (GO) and Kyoto Encyclopedia of Genes and Genomes (KEGG) pathway enrichment, complemented by protein interaction network analysis through the STRING database. For metabolomics, multivariate analyses, including orthogonal partial least squares‐discriminant analysis (OPLS‐DA), hierarchical clustering heatmaps and KEGG pathway annotation, were conducted in MetaboAnalyst 5.0. Group differences in metabolite levels were assessed with the Wilcoxon rank‐sum test.

For feature selection, five machine learning algorithms were applied: elastic‐net logistic regression; correlation‐based feature selection (CFS); the Boruta algorithm (maxRuns = 500); recursive feature elimination (RFE); and support vector machine (SVM). Each selected feature was assigned a weight based on its inclusion in each algorithm, and the sum of weights across all five methods was used to determine the final feature ranking. For subsequent diagnosis of sarcopenia, six machine learning algorithms—random forest (RF), support vector machine (SVM), least absolute shrinkage and selection operator (LASSO) regression, Gaussian naïve Bayes, gradient boosting machine (GBM) and K‐nearest neighbours (KNN)—were implemented as binary classifiers. The area under the receiver operating characteristic curve (AUC) of the models was reported as the median AUC after 10‐fold cross validation (CV). The sensitivity, specificity, precision and F1 score metrics were calculated at the optimal threshold. These analyses were performed using R version 4.2.3. Throughout this study, a *p*‐value less than 0.05 was considered statistically significant.

## 3 Results

### 3.1 Characteristics of the Participants

In this retrospective study, a total of 140 participants were recruited: 80 in the discovery cohort (40 sarcopenic and 40 non‐sarcopenic) and 60 in the validation cohort (30 sarcopenic and 30 non‐sarcopenic). The overall workflow is shown in Figure 1, and the enrollment flowchart is presented in Figure 2. Matched comparisons confirmed that age and sex were well balanced between sarcopenic and non‐sarcopenic groups in both cohorts (discovery: median 72.00 vs. 71.50 years, *p* = 0.714; validation: 71.00 vs. 71.50 years, *p* = 0.594; proportions of females 52.50% and 53.33% in the discovery and validation cohorts, respectively). Baseline characteristics of the sarcopenic (S) and non‐sarcopenic (NS) groups in the discovery and validation cohorts are summarized in Table 1. There were no statistically significant differences between S and NS groups in age, sex or the prevalence of age‐related comorbidities (hypertension, coronary heart disease, stroke and diabetes; all *p* > 0.05). As expected, the sarcopenic group exhibited significantly lower skeletal muscle mass index, grip strength and gait speed than the non‐sarcopenic group (all *p* < 0.05), consistent with the diagnostic criteria for sarcopenia and supporting the validity of our case–control stratification.

**TABLE 1:** Demographic and clinical characteristics in discovery cohort and validation cohort at baseline.

		Discovery cohort			Validation cohort		
		NS (*n* = 40)	S (*n* = 40)	*p*	NS (*n* = 30)	S (*n* = 30)	*p*
Age, median [IQR] (years)		72.00 [68.00, 77.00]	71.50 [69.00, 78.00]	0.7142	71.00 [67.00, 73.50]	71.50 [66.00, 74.75]	0.5936
Sex, female (%)		21 (52.50)	21 (52.50)	1.0000	16 (53.33)	16 (53.33)	1.0000
BMI, median [IQR] (kg/m^2^)		25.08 [24.15, 26.57]	21.36 [19.89, 22.84]	< 0.0001	24.66 [23.14, 26.57]	23.41 [21.46, 24.74]	0.0191
SMI, median [IQR] (kg/m^2^)	Female	6.00 [5.90, 6.40]	4.80 [4.60, 5.00]	< 0.0001	5.90 [5.90, 6.10]	5.45 [5.38, 5.50]	< 0.0001
	Male	7.20 [7.20, 7.30]	6.20 [5.75, 6.40]	< 0.0001	7.30 [7.20, 7.38]	6.60 [6.50, 6.70]	< 0.0001
Grip strength, median [IQR] (kg)	Female	22.60 [19.90, 25.20]	14.30 [13.70, 16.10]	< 0.0001	23.95 [20.48, 24.60]	16.15 [14.35, 16.92]	< 0.0001
	Male	35.20 [32.50, 38.30]	19.90 [17.75, 24.30]	< 0.0001	36.00 [33.82, 40.48]	23.85 [20.70, 25.65]	< 0.0001
Gait speed, median [IQR] (m/s)		1.25 [1.17, 1.32]	0.82 [0.75, 0.90]	< 0.0001	1.21 [1.11, 1.33]	0.83 [0.74, 0.91]	< 0.0001
Calf circumference, median [IQR] (cm)		33.80 [32.77, 35.85]	29.60 [28.17, 31.05]	< 0.0001	34.30 [32.92, 35.10]	32.15 [31.20, 33.20]	0.0010
Mid‐upper arm circumference, median [IQR] (cm)		28.05 [26.28, 29.42]	22.90 [21.58, 24.85]	< 0.0001	27.70 [25.80, 29.00]	24.35 [23.60, 25.75]	< 0.0001
Waist circumference, median [IQR] (cm)		91.20 [85.17, 96.58]	78.65 [73.05, 85.12]	< 0.0001	89.50 [83.50, 95.47]	84.15 [78.65, 89.42]	0.0133
Hip circumference, median [IQR] (cm)		95.40 [93.00, 99.72]	88.35 [84.95, 91.05]	< 0.0001	95.00 [92.45, 99.38]	90.70 [87.42, 93.38]	0.0003
Triceps skinfold thickness, median [IQR] (mm)		25.50 [19.73, 30.88]	21.20 [15.40, 25.12]	0.0278	26.50 [21.12, 33.38]	25.00 [16.25, 29.10]	0.1411
Hypertension (%)		13 (32.50)	8 (20.00)	0.3095	14 (46.67)	8 (26.67)	0.1799
Coronary heart disease (%)		0 (0.00)	2 (5.00)	0.4937	2 (6.67)	2 (6.67)	1.0000
Stroke (%)		0 (0.00)	5 (12.50)	0.0547	0 (0.00)	3 (10.00)	0.2373
Diabetes (%)		4 (10.00)	2 (5.00)	0.6752	6 (20.00)	2 (6.67)	0.2542

### 3.2 Proteomic Profiling Identified Sarcopenia‐Associated Inflammatory Signatures

**FIGURE 3:**
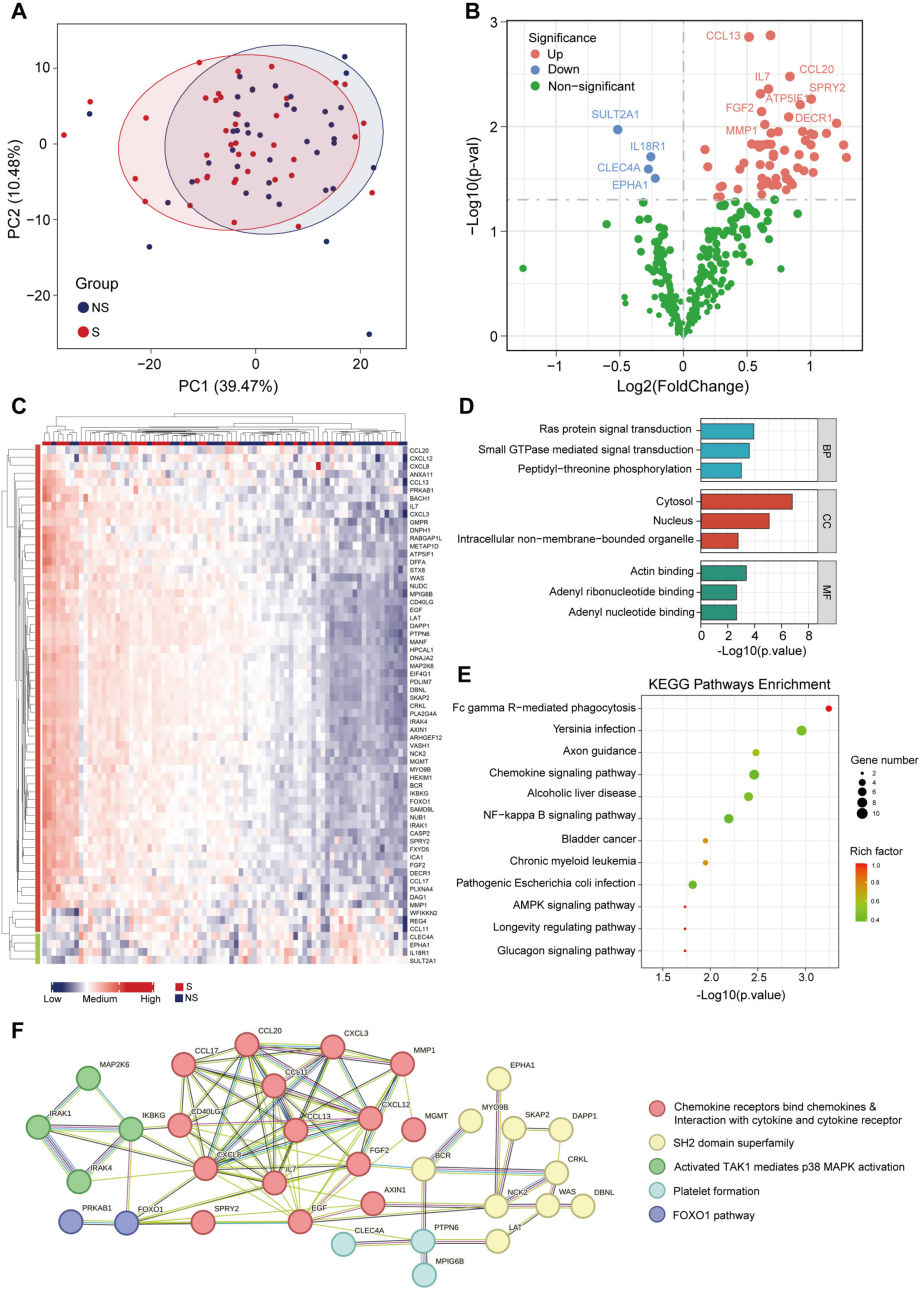
Proteomic profiles in sarcopenia and the analysis of differentially expressed proteins. (A) Principal component analysis (PCA) plot of differentially expressed proteins. (B) Volcano plot of differential proteins in plasma sample. Blue dots represent downregulated proteins; red dots represent upregulated proteins. (C) Heatmap of 65 differentially expressed proteins in discovery cohort. (D) Bar graph of Gene Ontology (GO) functional classification of differentially expressed proteins. (E) Bubble plots for Kyoto Encyclopedia of Genes and Genomes (KEGG) pathways enrichment of differentially expressed proteins. (F) Protein–protein interaction (PPI) network of differentially expressed proteins.

The plasma inflammatory protein profile was explored in sarcopenic and non‐sarcopenic subjects using the Olink 384 Inflammation Panel (Table S1). Principal component analysis (PCA) demonstrated distinct clustering patterns between groups in the discovery cohort (Figure 3A). As shown in the volcano plot and heatmap (Figure 3B,C), 65 differentially expressed proteins could be identified in sarcopenic patients (61 upregulated, 4 downregulated) (Table S2). GO analysis revealed that these proteins are enriched in several biological processes, including RAS protein and transduction, Small GTPase mediated signal transduction, and peptidyl‐threonine phosphorylation (Figure 3D). KEGG analysis of the differentially expressed proteins demonstrated enrichment of the Fc gamma R‐mediated phagocytosis, chemokine signalling and NF‐kappa B signalling pathways (Figure 3E). PPI network analysis revealed that the differentially expressed proteins were predominantly involved in chemokine and cytokine protein–protein interactions, further elucidating the association between sarcopenia and inflammation at the proteomic level (Figure 3F).

**FIGURE 4:**
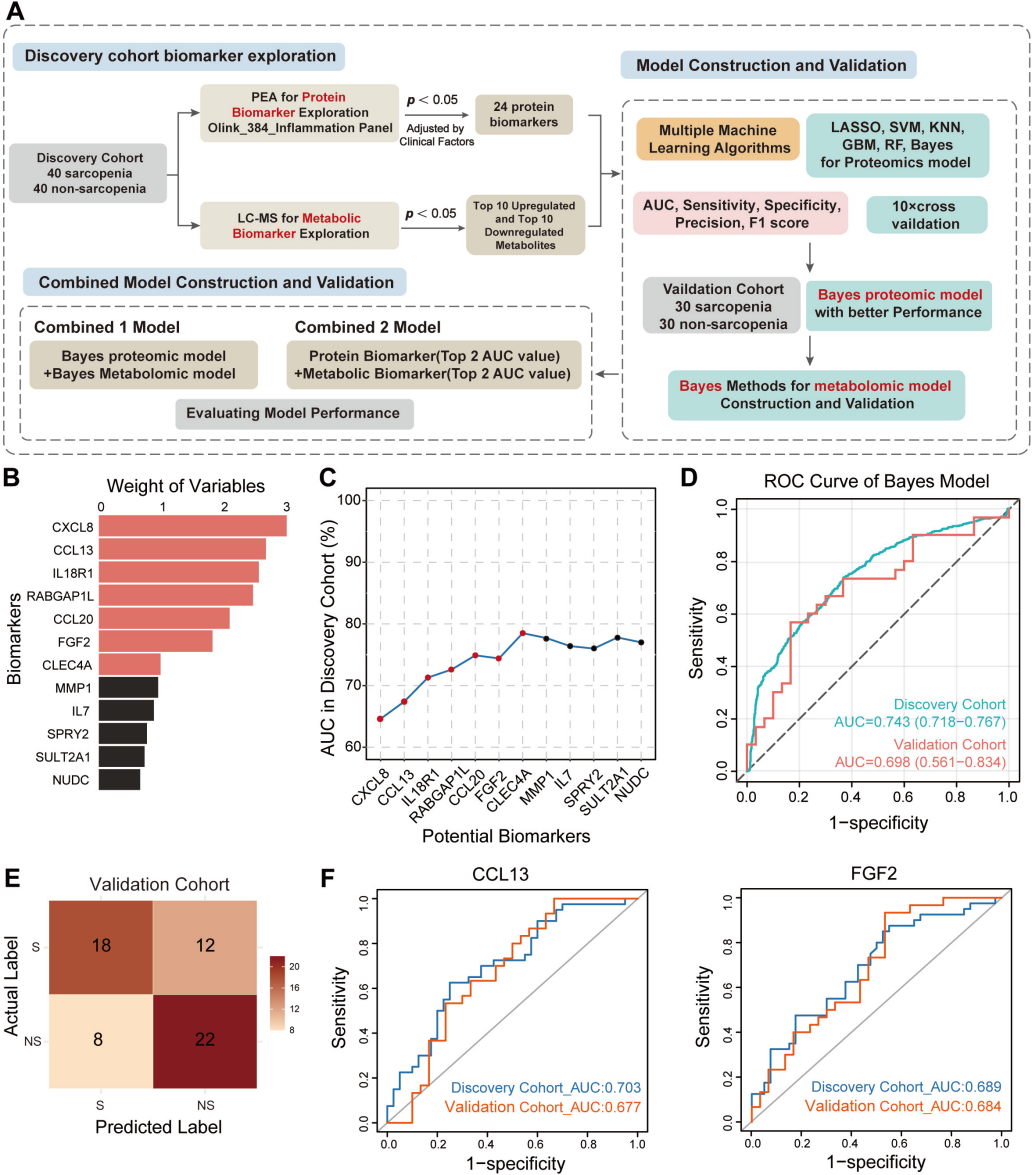
Proteomic‐based distinguish of sarcopenic (S) and non‐sarcopenic (NS) group by machine learning algorithms. (A) Workflow of biomarker discovery and model construction. (B) Bar chart of the top 12 protein features prioritized by the model. (C) Line chart of the change in area under the receiver operating characteristic curve (AUC) values after sequentially including variables. (D) Receiver operating characteristic (ROC) curves of Gaussian naïve Bayes model in discovery and validation cohorts. (E) Confusion matrix analysis of protein biomarkers in S versus NS group of validation cohorts. (F) ROC curves of CCL13 and FGF2 in discovery and validation cohorts.

To establish robust diagnostic models, we applied a multi‐algorithm machine learning framework with 10‐fold cross‐validation in the discovery cohort, and we subsequently evaluated the models using the independent validation cohort. The model construction workflow is illustrated in Figure 4A. First, we performed multivariable logistic regression adjusted for potential clinical covariates, including sex, age and BMI. Using this process, we identified 24 sarcopenia‐associated protein biomarkers (Figure S1). These protein biomarkers were then ranked by importance for subsequent model construction (Figure 4B). Next, six machine learning diagnostic models were constructed, incorporating feature selection based on variable importance. The AUCs of all machine learning models exceeded 0.70 in the discovery cohort and 0.55 in the validation cohort, demonstrating the potential diagnostic value of the inflammatory protein biomarkers (Figure S2 and Table S3). Among the machine learning models, the Gaussian naïve Bayes model demonstrated optimal diagnostic stability across both cohorts, achieving superior performance with an AUC of 0.698 (95% CI: 0.561–0.834) in the validation cohort through the integration of seven protein biomarkers (CXCL8, CCL13, IL18R1, RABGAP1L, CCL20, FGF2 and CLEC4A) (Figure 4B–D). The confusion matrix for the validation cohort demonstrated a sensitivity of 60.0% and a specificity of 73.3% (Figure 4E and Table 2). Next, 11 protein biomarkers were employed to develop six machine learning models, all of which showed significant differential expression in sarcopenia (Figure S3). Spearman's correlation analysis revealed potential associations between most of the protein signatures and two sarcopenia‐related clinical indicators (SMI and grip strength) (Figures S4 and S5). Individual biomarker evaluation identified CCL13 and FGF2 as the top‐performing biomarkers, with AUC values of 0.703 and 0.689 (respectively) in the discovery cohort and 0.677 and 0.684 (respectively) in the validation cohort (Figures 4F and S6). The observed differences in CCL13 and FGF2 levels between the sarcopenic and non‐sarcopenic groups were subsequently investigated using ELISA and confirmed to be significant (Figure S7).

**TABLE 2:** Diagnostic performance of proteomic, metabolomic, and Combined 1 and 2 models.

	Discovery cohort					Validation cohort				
	AUC (95% CI)	Sensitivity	Specificity	Precision	F1	AUC (95% CI)	Sensitivity	Specificity	Precision	F1
Proteomic	0.743 (0.718–0.767)	0.608	0.750	0.708	0.654	0.69 (0.561–0.834)	0.600	0.733	0.692	0.643
Metabolomic	0.828 (0.808–0.849)	0.414	0.905	0.813	0.548	0.751 (0.617–0.885)	0.433	0.900	0.812	0.565
Combined 1	0.951 (0.937–0.965)	0.905	0.850	0.858	0.881	0.823 (0.717–0.930)	0.633	0.900	0.864	0.731
Combined 2	0.853 (0.828–0.878)	0.818	0.705	0.735	0.774	0.911 (0.839–0.983)	0.867	0.800	0.812	0.839

### 3.3 Metabolomics Analysis of Sarcopenic Patients

**FIGURE 5:**
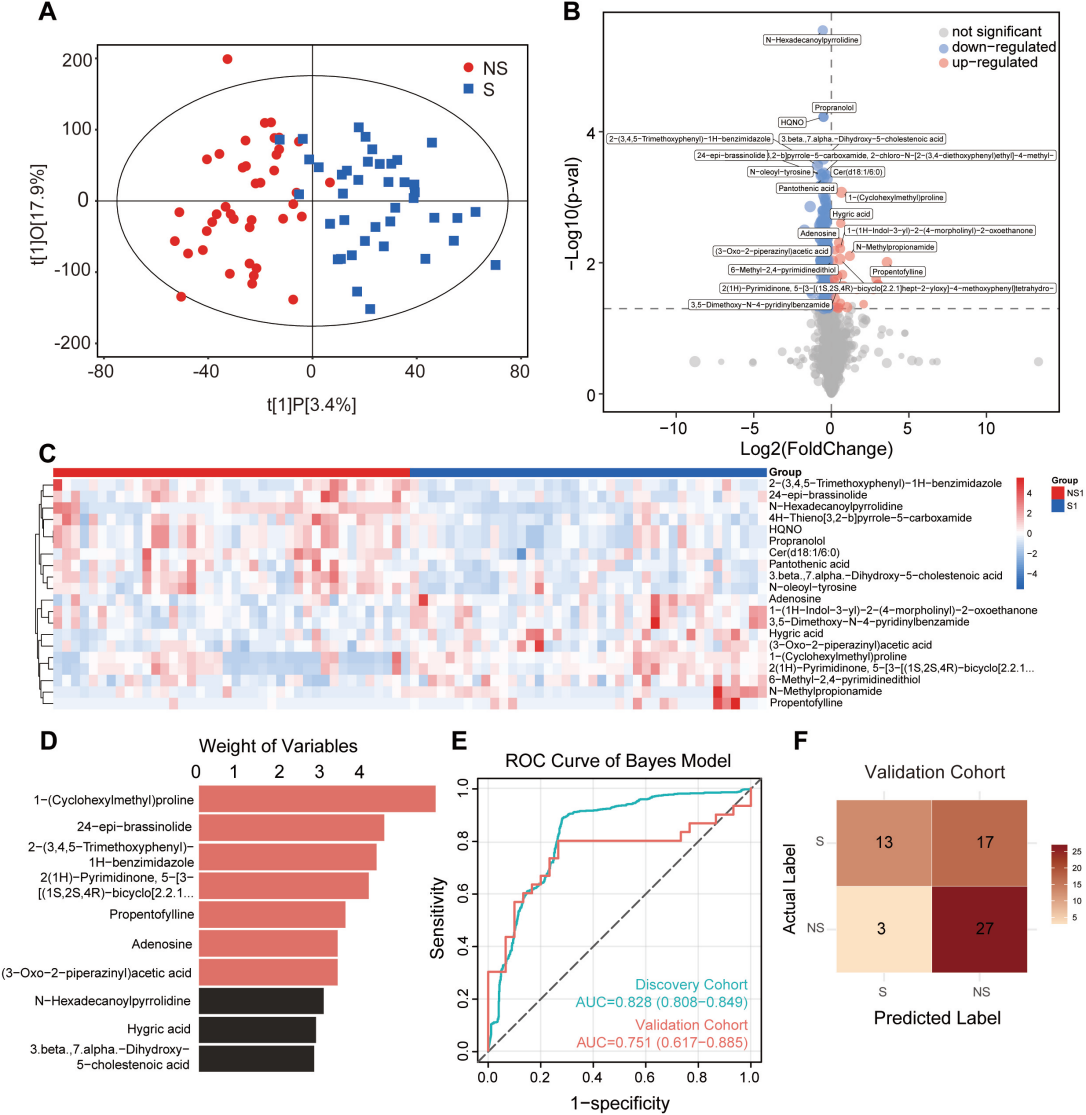
Metabolic profiles in sarcopenic and non‐sarcopenic group. (A) Orthogonal partial least squares–discriminant analysis (OPLS–DA) score plot for S and NS group. (B) Volcano plot of differential metabolites between S and NS. (C) Heatmap of top 20 differential metabolites in discovery cohort. (D) Feature importance ranking of the top 10 metabolites in the Gaussian naïve Bayes model. (E) ROC curves of the Bayes metabolomic model in the discovery and validation cohorts. (F) Confusion matrix analysis of metabolism biomarkers in S versus NS group of validation cohort.

Orthogonal partial least squares‐discriminant analysis (OPLS‐DA) revealed significant metabolic discrimination between the two groups (Figure 5A). Untargeted metabolomic profiling revealed 268 significantly altered metabolites in the sarcopenic group compared with controls, including 28 upregulated and 240 downregulated metabolites (Figure 5B and Table S4). The top 10 upregulated metabolites and top 10 downregulated metabolites distinguishing sarcopenia from non‐sarcopenic cohorts were highlighted in a heatmap (Figure 5C). KEGG Pathway enrichment analysis identified three core dysregulated metabolic pathways (Figure S8), with the glycine‐serine–threonine metabolism, branched‐chain amino acid biosynthesis, and amino acid biosynthesis pathways all showing significant enrichment scores (> 3).

Building on the analytical consistency observed in our proteomic study, we applied the same Bayes algorithm to evaluate the diagnostic potential of metabolic features. We ranked the 20 selected metabolites (top 10 upregulated and top 10 downregulated) by variable importance and sequentially incorporated them into the Bayes metabolic model (Figure 5D). The Bayes algorithm optimized a subset of seven metabolic features with robust diagnostic performance, achieving an AUC of 0.828 (95% CI: 0.808–0.849) in the discovery cohort and an AUC of 0.751 (95% CI: 0.617–0.885) in the independent validation cohort (Figure 5E). An analysis of diagnostic performance using the confusion matrix revealed a sensitivity of 43.3% and a specificity of 90.0% (Figure 5F and Table 2). A differential boxplot of the top 10 most important metabolites is presented in Figure S9. Additionally, two key metabolites—1‐(cyclohexylmethyl)proline and N‐hexadecanoylpyrrolidine—were identified from analyses of the diagnostic performance of individual metabolites, with AUCs of 0.693 and 0.782 (respectively) in the discovery cohort and AUCs of 0.828 and 0.888 (respectively) in the validation cohort (Figure S10).

### 3.4 Combined Multi‐Omics Models Outperformed the Single‐Omics Approaches

Given the suboptimal diagnostic performance of single‐omics approaches, we developed two multimodal integration strategies for sarcopenia diagnosis. Combined Model 1 was constructed by integrating the proteomic and metabolomic Bayes models using logistic regression. This multi‐omics approach demonstrated superior performance with an AUC of 0.951 (95% CI: 0.937–0.965) in the discovery cohort, significantly outperforming single‐omics models (proteomic: *p* < 0.001; metabolomic: *p* < 0.001). The diagnostic advantage persisted in the validation cohort, with Combined Model 1 outperforming the proteomic model (AUC: 0.823 vs. 0.698, *p* = 0.037), while exhibiting comparable performance to the metabolomic model (AUC: 0.823 vs. 0.751, *p* = 0.147) (Figure S11A,B).

**FIGURE 6:**
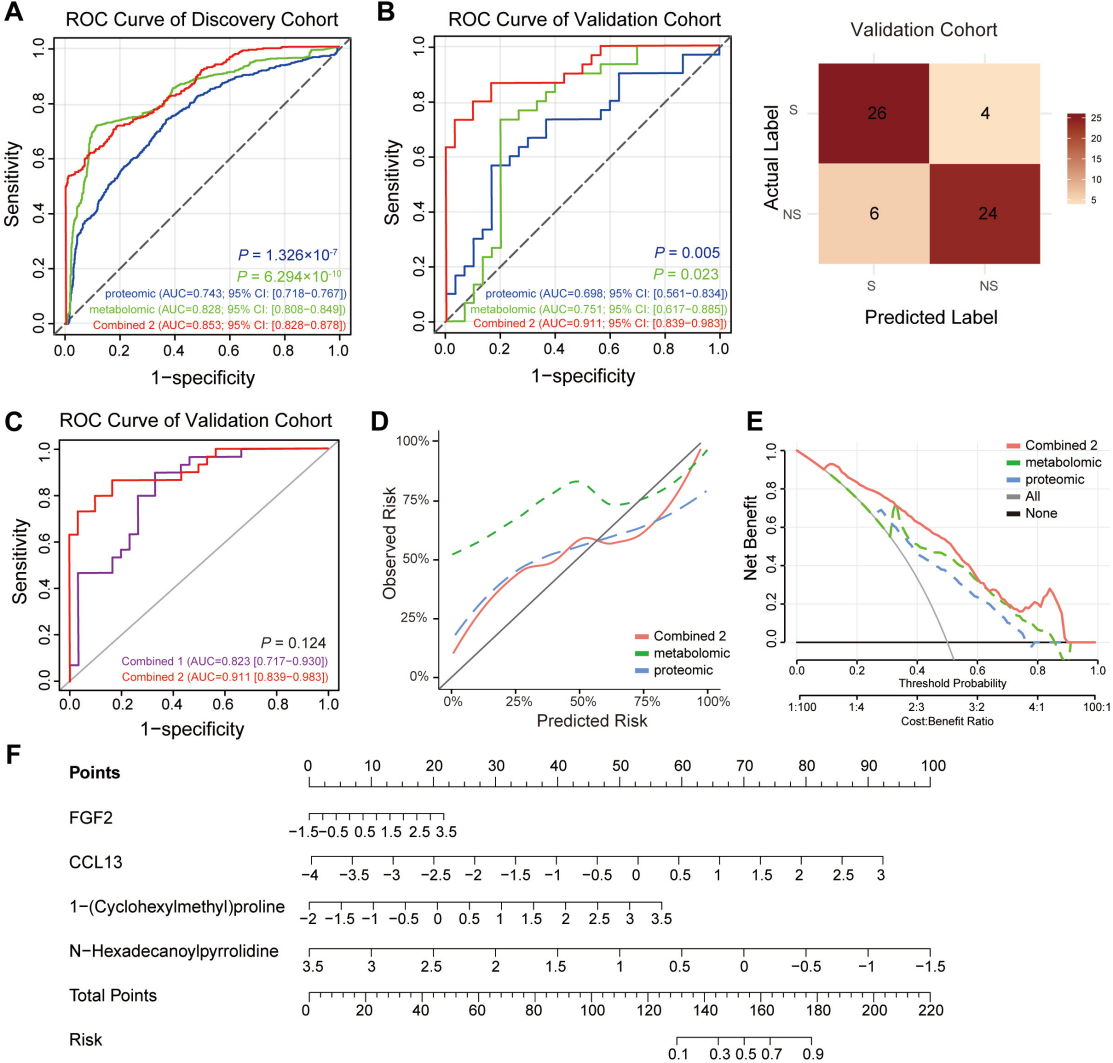
Assessment of the prediction ability of proteomic, metabolomic models and Combined Model 2 for sarcopenia. (A) ROC curves for proteomic, metabolomic models and Combined Model 2 in discovery cohort. (B) ROC curves for proteomic, metabolomic models and Combined Model 2 in the validation cohort, with confusion matrix for Combined Model 2. (C) ROC curves for Combined Model 1 and 2 in validation cohort. (D) Calibration curves of proteomic, metabolomic models and Combined Model 2 predicting the probability of sarcopenia in discovery cohort. (E) Decision curve analysis for proteomic, metabolomic models and Combined Model 2 in the discovery cohort. (F) Nomogram for the prediction of sarcopenia in the overall population.

To improve clinical applicability, Combined Model 2 was constructed by integrating the top two biomarkers from each omics platform: CCL13 and FGF2 from proteomics and 1‐(cyclohexylmethyl)proline and N‐hexadecanoylpyrrolidine from metabolomics. Despite the reduction in feature count, Combined Model 2 achieved equivalent validation performance to Combined Model 1 (AUC = 0.911 vs. 0.823, *p* = 0.124) (Figure 6C), while significantly surpassing the single‐omics models in both the discovery (proteomic: 0.853 vs. 0.743, *p* < 0.001; metabolomic: 0.853 vs. 0.828, *p* < 0.001) and validation cohorts (proteomic: 0.911 vs. 0.698, *p* = 0.005; metabolomic: 0.911 vs. 0.751, *p* = 0.023) (Figure 6A,B). In sensitivity analyses that explicitly added BMI as a covariate to Combined Model 2, model performances were materially unchanged (AUC: 0.936 vs. 0.911, *p* = 0.418), indicating the robustness of Combined Model 2 to BMI (Figure S12A). Age‐stratified analyses (< 70 vs. ≥ 70) likewise showed no significant AUC difference for Combined Model 2 (AUC: 0.924 vs. 0.883, *p* = 0.606), suggesting consistent performance across age strata (Figure S12C). Confusion matrices for the BMI‐adjusted and age‐stratified analyses are provided in Table S5 and Figure S12B,D.

An overall AUC comparison of the two combined models versus the single‐omics models is presented in Figure S11E. Confusion matrix analysis provided sensitivity, specificity, precision and F1 scores for each model. Both Combined Model 1 and Combined Model 2 exhibited stronger sensitivity and precision, as well as higher F1 scores than single‐omics models (Figures S11C and 6B and Table 2). Combined Model 2 achieved a sensitivity of 86.7%, a specificity of 80.0%, a precision of 81.2% and an F1 score of 0.839. Calibration analysis revealed that Combined Model 2 demonstrated enhanced agreement between predicted and observed outcomes (Figure 6D and Table S6). Additionally, a DCA curve revealed that the benefit of Combined Model 2 was higher than that of the proteomic or metabolomic model alone (Figure 6E). Subsequently, Combined Model 1 and Combined Model 2 were used to evaluate the sarcopenia risk of the participants. A nomogram integrating CCL13, FGF2, 1‐(cyclohexylmethyl)proline and N‐hexadecanoylpyrrolidine is shown in Figure 6F. Additionally, a nomogram showing Combined Model 1 is shown in Figure S11D.

## 4 Discussion

While the use of plasma biomarkers for the diagnosis of aging‐related conditions shows promise, robust diagnostic models for sarcopenia are still lacking. Current approaches primarily rely on single‐omics strategies or individual biomarkers, which are susceptible to confounding by comorbidities, medications, environmental exposures, and inter‐individual variability [15]. A recent meta‐analysis assessing a total of 30 biomarkers has revealed that existing single‐omics biomarkers are not optimal for screening or diagnosing sarcopenia because of their low‐to‐moderate accuracy [7]. While multi‐omics integration holds potential for uncovering sarcopenia's complex pathophysiology, its application has been largely confined to mechanistic studies or target discovery, with limited focus on developing clinically applicable diagnostic models [16, 17]. While one previous study has characterized plasma proteomic‐metabolomic profiles in individuals with primary sarcopenia and end‐stage renal disease‐related muscle wasting, it did not establish machine learning models nor did it define AUC and precision metrics for biomarkers [18]. Furthermore, existing applications of machine learning in sarcopenia research predominantly focused on single‐modal data or conventional clinical parameters and were hampered by these limitations. For instance, a prior study employing machine learning algorithms on isolated proteomic data lacked validation in independent cohorts [19]. Zhang et al. used clinical characteristics and laboratory markers for the prediction of sarcopenia based on machine learning, but the sensitivity of their highest‐efficiency model was only 0.524 [20].

To address these issues, our study presents a novel machine learning‐driven framework integrating Olink proteomics (384 inflammation panel) and LC–MS‐based untargeted metabolomics for sarcopenia diagnosis. To our knowledge, this is the first study to combine the two advanced platforms within a machine learning pipeline to build diagnostic models for sarcopenia. Crucially, our results demonstrate that integrating proteomic and metabolomic data significantly enhances diagnostic accuracy compared to relying on either modality alone. Both of the combined models we developed significantly outperformed the single‐omics models across various metrics, including AUC, sensitivity, specificity, and precision. Our Combined Model 1 integrated the probabilistic outputs of the best‐performing proteomic (Gaussian naïve Bayes, 7 proteins) and metabolomic (Gaussian naïve Bayes, 7 metabolites) models via logistic regression, achieving an AUC exceeding 0.95 in the discovery cohort and over 0.8 in the validation cohort. Seeking greater clinical utility and scalability, we next developed Combined Model 2, which utilized only the top two prioritized biomarkers from each platform. Remarkably, despite its simplicity, Combined Model 2 achieved outstanding validation performance, significantly surpassing both the proteomic model (AUC 0.911 vs. 0.698, *p* = 0.005) and the metabolomic model (AUC 0.911 vs. 0.751, *p* = 0.023), while demonstrating comparable efficacy to the more complex Combined Model 1 (AUC 0.911 vs. 0.823, *p* = 0.124). Indeed, Combined Model 2 achieved a sensitivity of 86.7%, a specificity of 80.0%, a precision of 81.2% and an F1 score of 0.839 in the validation cohort. In sensitivity analyses, Combined Model 2 also demonstrated robustness to BMI and yielded similar AUCs across age strata, supporting stability across patient subgroups. An atypical but noteworthy observation is that Combined Model 2 validated with a higher AUC than in discovery; similar phenomena have been reported [20, 21] and are plausibly attributable to greater between‐group separability of the four biomarkers in the validation cohort and the relatively small sizes of our cohorts.

Notably, the diagnostic performances of our combined models, and especially that of the efficient and clinically practical four‐biomarker panel (Combined Model 2), exceeded the diagnostic performance of all currently reported single biomarkers or biomarker panels for sarcopenia, including proteomic markers MMP9/TIMP1 [22] and RBP [23], and metabolic markers such as Cr/CysC [24, 25], AST/ALT [26] and Irisin [27].

Beyond diagnostics, our multi‐omics profiling offers valuable pathophysiological insights into inflammation and metabolic dysfunction in sarcopenia. Using the inflammation‐focused Olink panel, we identified 65 dysregulated proteins, and these were significantly enriched in important inflammation pathways, including Fc gamma R‐mediated phagocytosis and chemokine signalling, reinforcing the established role of chronic inflammation in sarcopenia. Complementing the proteomic findings, metabolomic analysis detected 268 dysregulated metabolites, with pathway enrichment highlighting profound disturbances in amino acid metabolism, specifically glycine‐serine‐threonine metabolism and branched‐chain amino acid (BCAA; leucine, isoleucine, valine) biosynthesis. These findings confirm the critical role played by amino acids, especially BCAAs, as signalling molecules activating muscle protein synthesis, and support recent evidence linking impaired BCAA catabolism to sarcopenia [28].

Convergent biological evidence supports the plausibility of the prioritized biomarkers comprising Combined Model 2. CCL13, an inflammation‐linked chemokine, has been observed to be elevated in the plasma of individuals suffering from aging‐related cognitive impairment, and it demonstrates moderate diagnostic efficacy in Alzheimer's disease (ad) [29]. In our study, all participants underwent standardized cognitive screening at enrollment, and those with ad were excluded; therefore, higher CCL13 likely reflects sarcopenia‐related inflammatory activity rather than ad‐driven effects, supporting the inclusion of CCL13 within a multi‐marker panel for sarcopenia. FGF2, a growth factor, is involved in muscle repair, and it is increased in aged muscle. Moreover, FGF2 has previously been explored as a methylation‐based biomarker [30]. N‐Hexadecanoylpyrrolidine is a fatty acid amide. This class of metabolites has been shown to exhibit strong associations with muscle mass and strength, suggesting that N‐hexadecanoylpyrrolidine is a promising candidate for use as a circulating biomarker for the early detection and monitoring of sarcopenia in older males [31]. 1‐(Cyclohexylmethyl)proline has been linked to dysregulated proline metabolism, an observation that supports findings implicating altered gut microbial proline metabolism in sarcopenia [32]. When combined together, this minimalist panel achieved high diagnostic performance (AUC > 0.9) by capturing inflammatory aging, metabolic stress and tissue repair dynamics, thereby outperforming conventional single‐marker approaches while maintaining clinical practicality. The exceptional performance and streamlined design of this four‐biomarker panel position it as a promising candidate for translation into a clinical diagnostic kit. The two protein biomarkers (CCL13, FGF2) are readily quantifiable using widely available immunoassay platforms (e.g., ELISAs), and the two metabolites (N‐hexadecanoylpyrrolidine, 1‐(cyclohexylmethyl)proline) can be efficiently measured using targeted LC–MS/MS. By restricting the panel to four analytes, assay complexity and cost are both significantly reduced compared to large‐scale omics profiling, greatly enhancing the potential scalability of this assay for population screening and routine clinical use.

While the combined proteomic‐metabolomic biomarkers identified in this study demonstrate high diagnostic performance, several limitations warrant consideration. First, sample size was modest. Independent validation in larger, multi‐centre cohorts is essential to confirm generalizability. Second, the absence of long‐term follow‐up data precludes assessment of biomarker stability over time or their predictive value for clinically relevant outcomes such as frailty progression. Future longitudinal studies that track these biomarkers alongside functional assessments should be performed to establish their prognostic utility. Third, the cohort was ethnically homogeneous—all participants were Han Chinese—which limits generalizability to other ethnic groups and non‐Asian populations that differ in body composition and sarcopenia prevalence; future validations across diverse ancestries and geographic settings are warranted.

In conclusion, the results presented in this study establish high‐performance, plasma‐based diagnostic models for sarcopenia by innovatively integrating inflammatory proteomic and metabolomic profiling within a machine learning framework. We demonstrate that multi‐omics integration significantly outperforms single‐omics approaches, providing a more comprehensive reflection of the disease's complexity. Crucially, we have identified a high‐performing, minimal four‐biomarker panel (CCL13, FGF2, N‐hexadecanoylpyrrolidine, 1‐(cyclohexylmethyl)proline) that offers exceptional diagnostic accuracy and strong potential for clinical translation due to its streamlined nature. Beyond diagnostics, our findings reinforce the notion that inflammatory dysregulation and metabolic dysfunction both play a role in sarcopenia pathogenesis, providing insights for further mechanistic exploration and targeted therapeutic strategies.

## Funding

This work is supported by grants from the National Key R&D Program of China (No. 2024YFE0104700), Sichuan Science and Technology Program, the Central Government Guides Local Science and Technology Development Projects, China (Grant No. 2024ZYD0065), the National Natural Science Foundation of China (No. 32090043), the National Clinical Research Center for Geriatrics, West China Hospital, Sichuan University (Z2024JC003, Z20201009), the 1·3·5 Project for Disciplines of Excellence, West China Hospital, Sichuan University (Grant No. ZYYC25009). The funders had no role in the study design, data collection, analysis, decision to publish or manuscript preparation.

## Ethics Statement

This study was conducted under approval by the Institutional Review Board of West China Hospital (Project identification code: 2019.791), with written informed consent secured from all participants. Sample handling protocols conformed to the ethical standards of the Declaration of Helsinki.

## Conflicts of Interest

The authors declare no conflicts of interest.

## Supporting information


**Figure S1:** Odds ratios of individual proteins for sarcopenia. (A) Odds ratios of each protein after adjustment for clinical covariates. (B) Odds ratios of the top 20 differentially expressed proteins without adjustment.
**Figure S2:** ROC curve of multiple machine learning Models in discovery cohort and validation cohort.
**Figure S3:** Venn plot depicting the overlap of proteins in six machine learning models. Box plots showing the expression of the 11 selected protein in S and NS in discovery cohort.
**Figure S4:** Correlation between the SMI and the expression levels of 11 individual proteins.
**Figure S5:** Correlation between the grip and the expression levels of 11 individual proteins.
**Figure S6:** ROC curve of 11 individual proteins.
**Figure S7:** Plasma protein levels of CCL13 and FGF2 measured by ELISA in the discovery cohort.
**Figure S8:** (A) Bubble plots for KEGG pathways enrichment of differential metabolites. (B) Pathway topology analysis metabolites that distinguished sarcopenia from non‐sarcopenia.
**Figure S9:** Box plots showing the expression of the top 10 differential metabolites in S and NS in discovery cohort.
**Figure S10:** ROC curve of the top 10 differential metabolites.
**Figure S11:** Assessment of the prediction ability of proteomic, metabolomic models and Combined Model 1 for sarcopenia. (A) ROC curves for proteomic, metabolomic models and Combined Model 1 in discovery cohort. (B) ROC curves for proteomic, metabolomic models and Combined Model 1 in validation cohort. (C) Confusion matrix analysis of Combined Model 1 in S versus NS group of validation cohort. (D) Nomogram for the prediction of sarcopenia in whole population. (E) Summary table of comparison values of AUC among groups.
**Figure S12:** Assessment of the model performance with BMI Augmentation and Age‐Based Stratification. (A) ROC curves for Combined 2 and Combined 2 + BMI models in validation cohort. (B) Confusion matrix analysis of Combined 2 + BMI model in S versus NS group of validation cohort. (C) ROC curves for the age‐stratified Combined 2 model in the validation cohort. (D) Confusion matrix analysis of Combined 2 (Age ≥ 70) (left) and Combined 2 (Age < 70) (right) models in S versus NS group of validation cohort.

**Table S1:** Olink Explore 384 protein list.
**Table S2:** Differential expression results of proteins
**Table S3:** Performance of six machine learning proteomic models.
**Table S4:** Differential expression results of metabolomics data.
**Table S5:** Diagnostic performance of Combined 2 + BMI, Combined 2 (age ≥ 70) and Combined 2 (age < 70) models.
**Table S6:** Calibration slope, intercept, and brier score of proteomic, metabolomic models and Combined 2 models.

## Data Availability

The data that support the findings of this study are available from the corresponding author upon reasonable request.
