# Supplementary material for: Integrated Proteomic and Metabolomic Profiling for Developing Novel Plasma‐Based Diagnostic Models of Sarcopenia
Source: J Cachexia Sarcopenia Muscle. 2026 Jan 16;17(1):e70188. doi: 10.1002/jcsm.70188 (PMC12811042; doi:10.1002/jcsm.70188)
Supplement: Supplementary file 1 — Figure S1: Odds ratios of individual proteins for sarcopenia. (A) Odds ratios of each protein after adjustment for clinical covariates. (B) Odds ratios of the top 20 differentially expressed proteins without adjustment. Figure S2: ROC curve of multiple machine learning Models in discovery cohort and validation cohort. Figure S3: Venn plot depicting the overlap of proteins in six machine learning models. Box plots showing the expression of the 11 selected protein in S and NS in discovery cohort. Figure S4: Correlation between the SMI and the expression levels of 11 individual proteins. Figure S5: Correlation between the grip and the expression levels of 11 individual proteins. Figure S6: ROC curve of 11 individual proteins. Figure S7: Plasma protein levels of CCL13 and FGF2 measured by ELISA in the discovery cohort. Figure S8: (A) Bubble plots for KEGG pathways enrichment of differential metabolites. (B) Pathway topology analysis metabolites that distinguished sarcopenia from non‐sarcopenia. Figure S9: Box plots showing the expression of the top 10 differential metabolites in S and NS in discovery cohort. Figure S10: ROC curve of the top 10 differential metabolites. Figure S11: Assessment of the prediction ability of proteomic, metabolomic models and Combined Model 1 for sarcopenia. (A) ROC curves for proteomic, metabolomic models and Combined Model 1 in discovery cohort. (B) ROC curves for proteomic, metabolomic models and Combined Model 1 in validation cohort. (C) Confusion matrix analysis of Combined Model 1 in S versus NS group of validation cohort. (D) Nomogram for the prediction of sarcopenia in whole population. (E) Summary table of comparison values of AUC among groups. Figure S12: Assessment of the model performance with BMI Augmentation and Age‐Based Stratification. (A) ROC curves for Combined 2 and Combined 2 + BMI models in validation cohort. (B) Confusion matrix analysis of Combined 2 + BMI model in S versus NS group of validation cohort. (C) ROC curves [file JCSM-17-e70188-s002.docx]

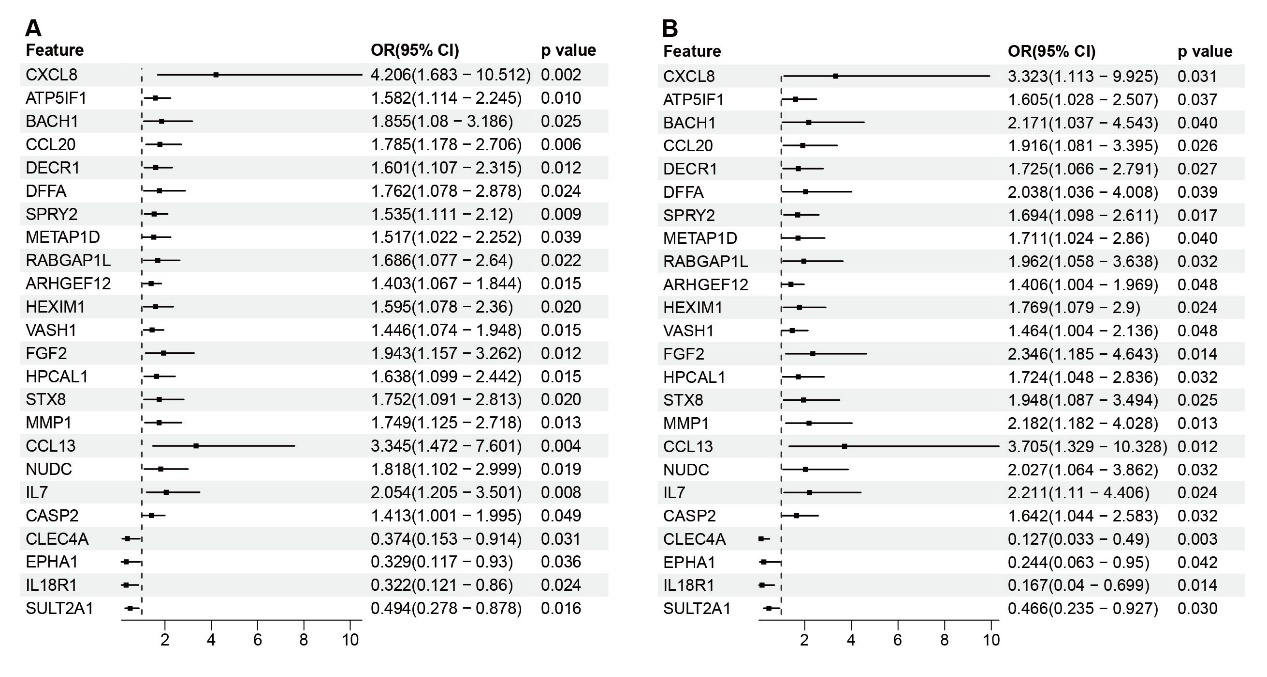


Fig. S1 Odds ratios of individual proteins for sarcopenia.
(A) Odds ratios of each protein after adjustment for clinical covariates.
(B) Odds ratios of the top 20 differentially expressed proteins without adjustment.


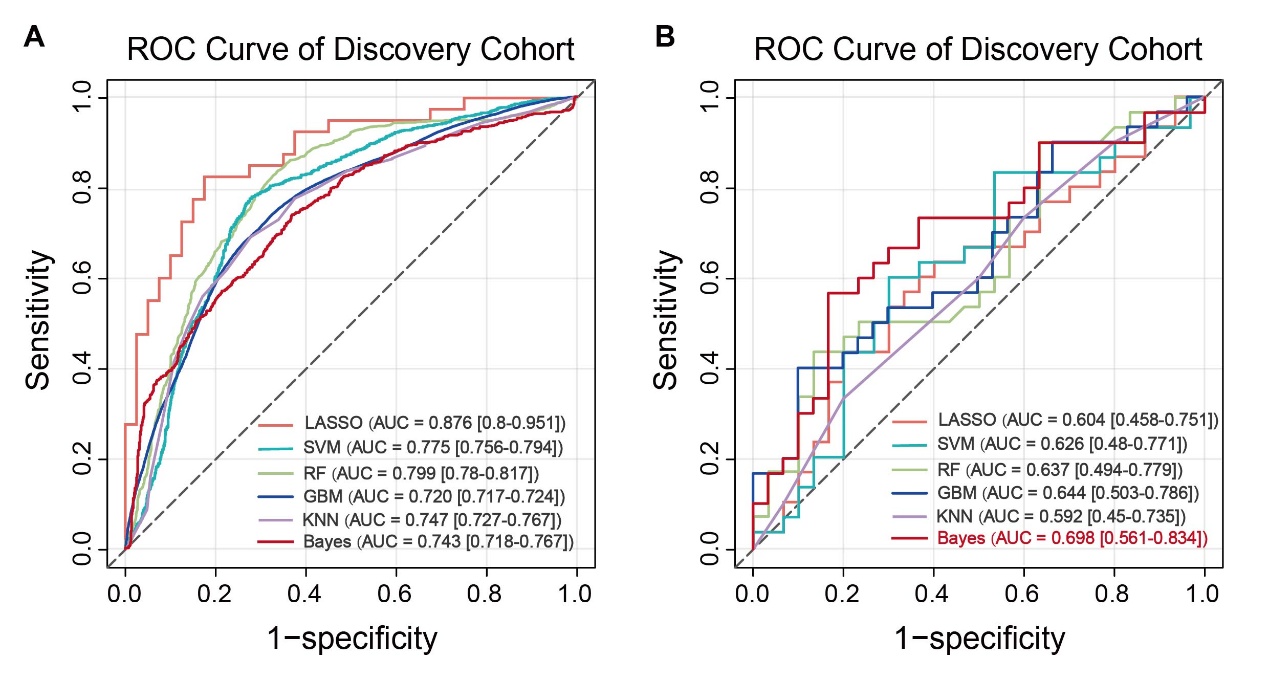


Fig. S2 ROC curve of multiple machine learning Models in discovery cohort and validation cohort.


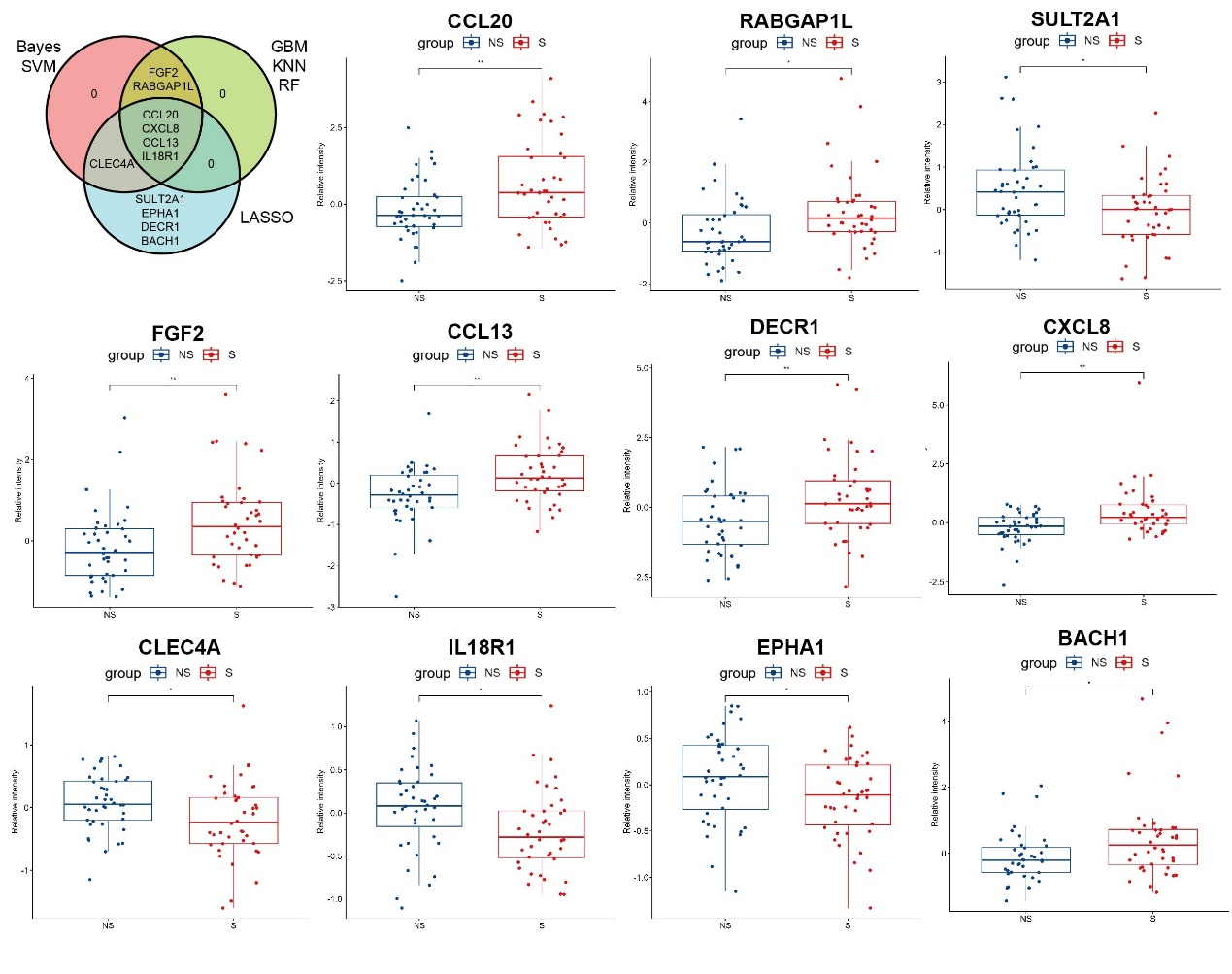


Fig. S3 Venn plot depicting the overlap of proteins in six machine learning models. Box plots showing the expression of the 11 selected protein in S and NS in discovery cohort.


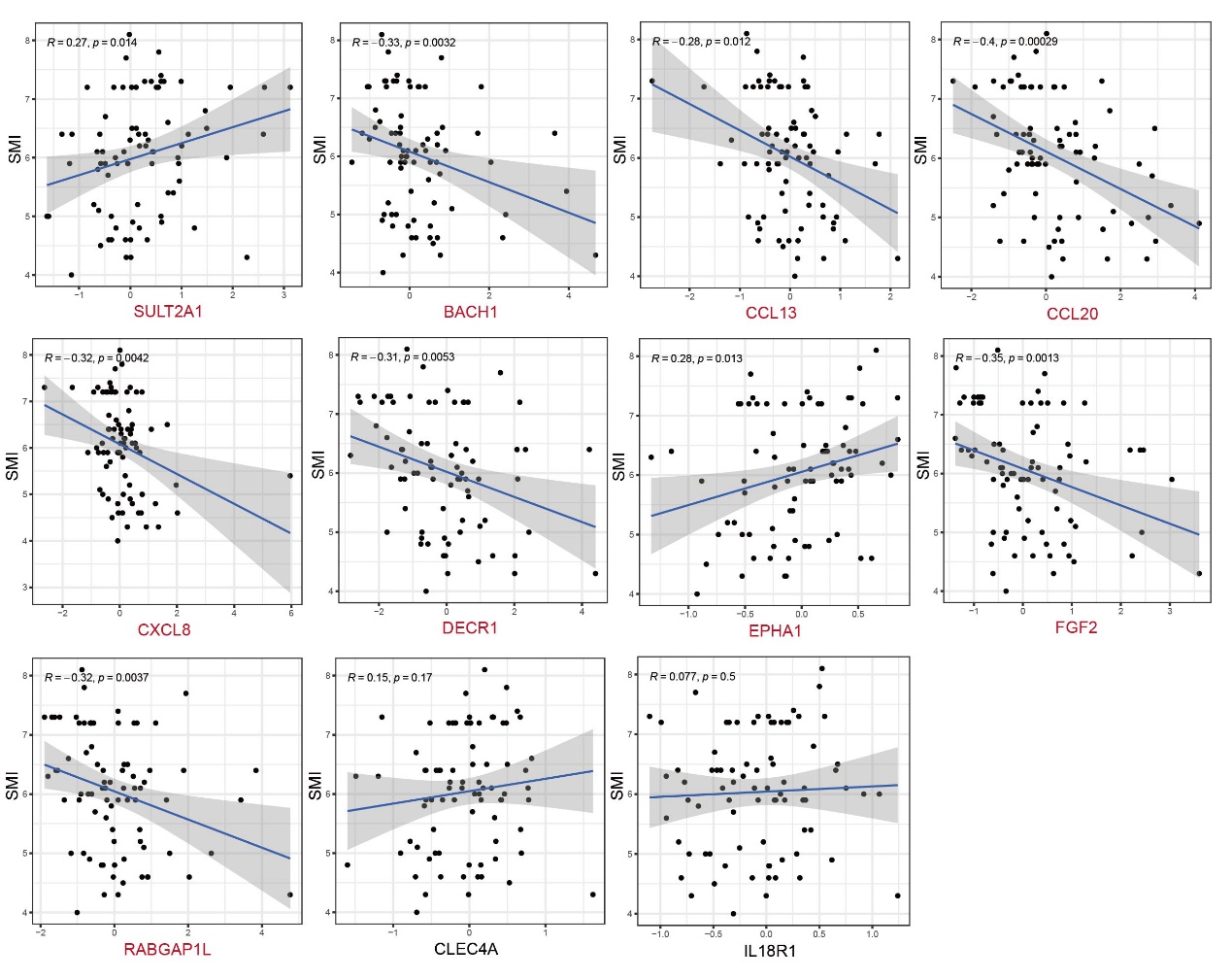


Fig. S4 Correlation between the SMI and the expression levels of 11 individual proteins


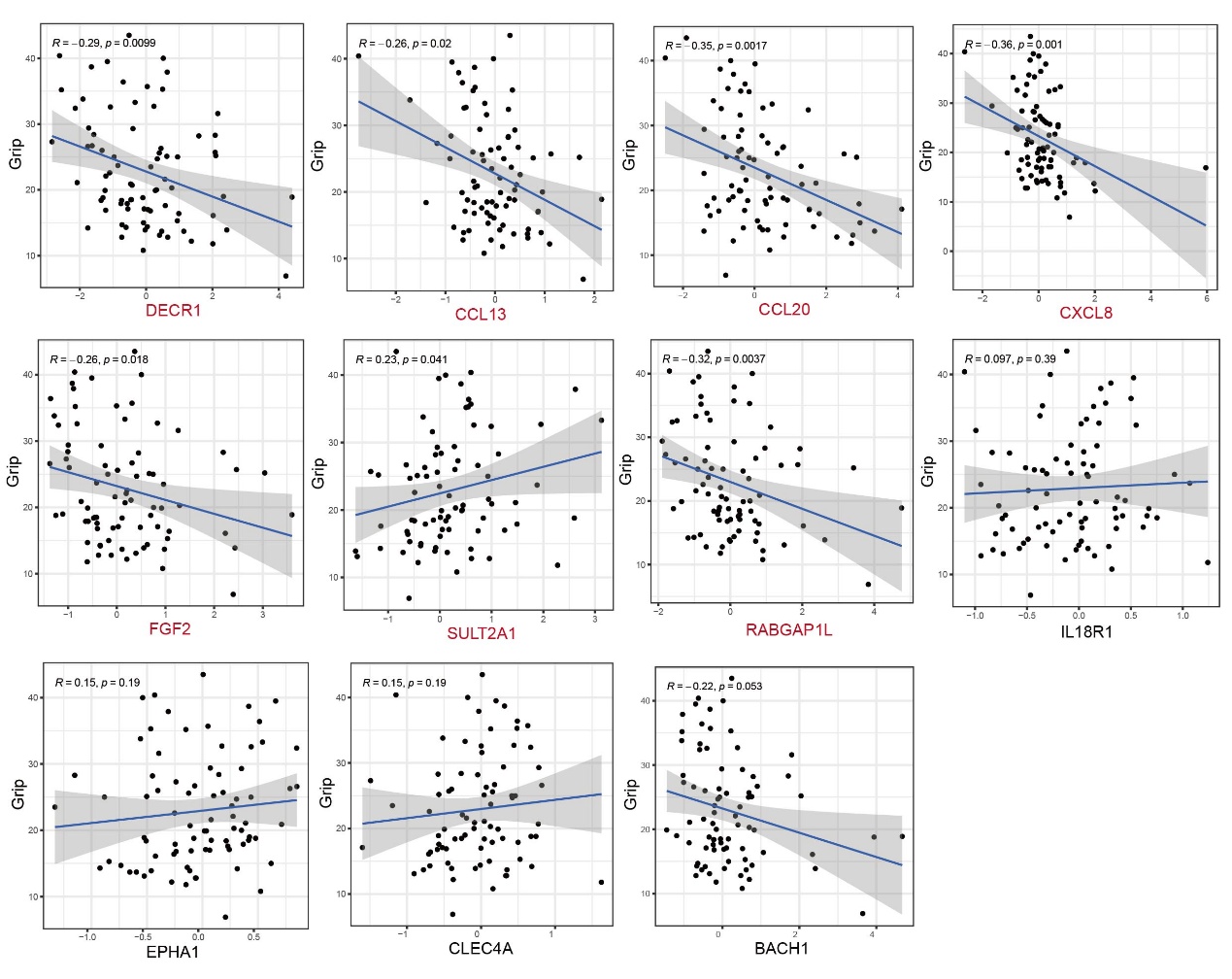


Fig. S5 Correlation between the grip and the expression levels of 11 individual proteins


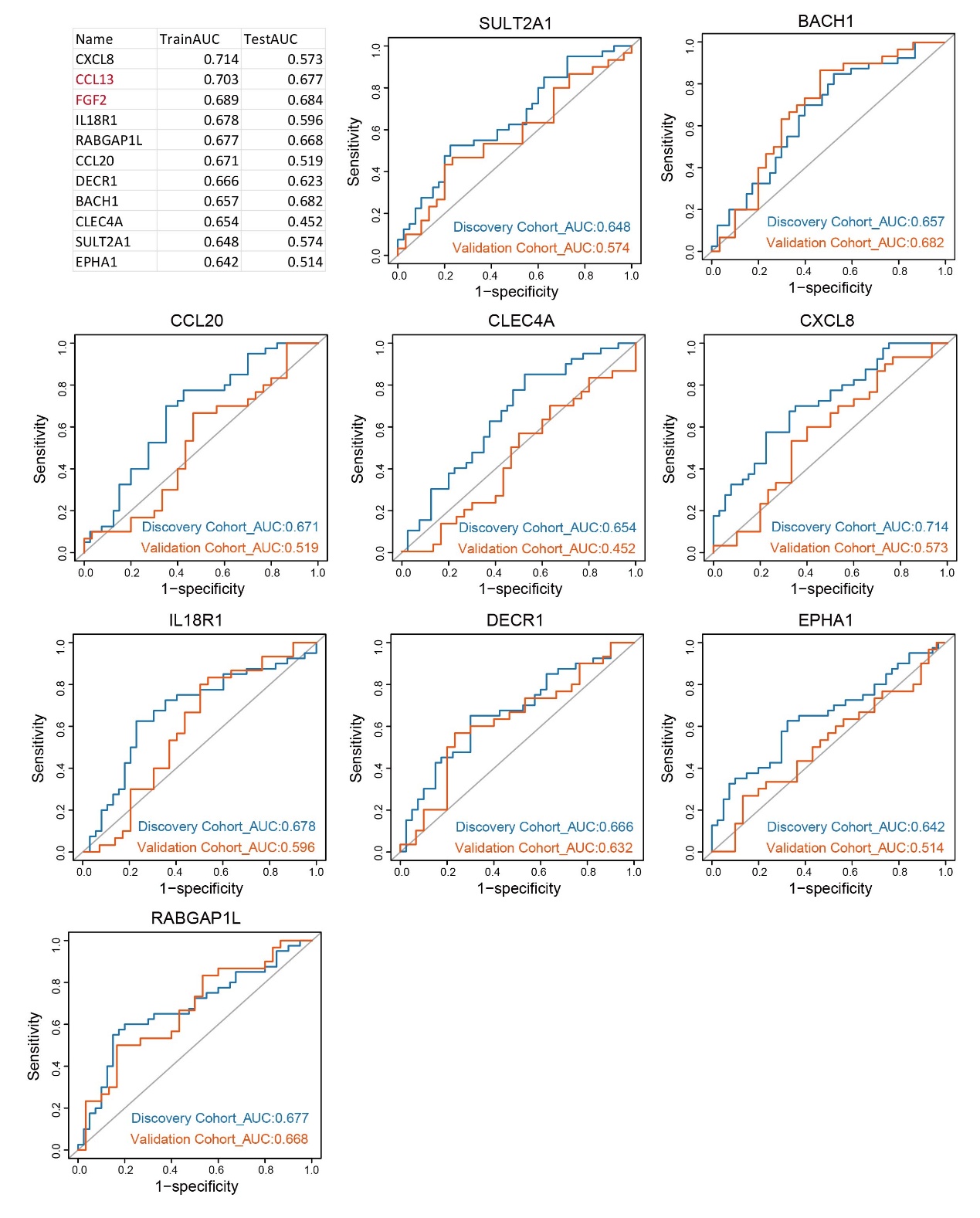


Fig. S6 ROC curve of 11 individual proteins


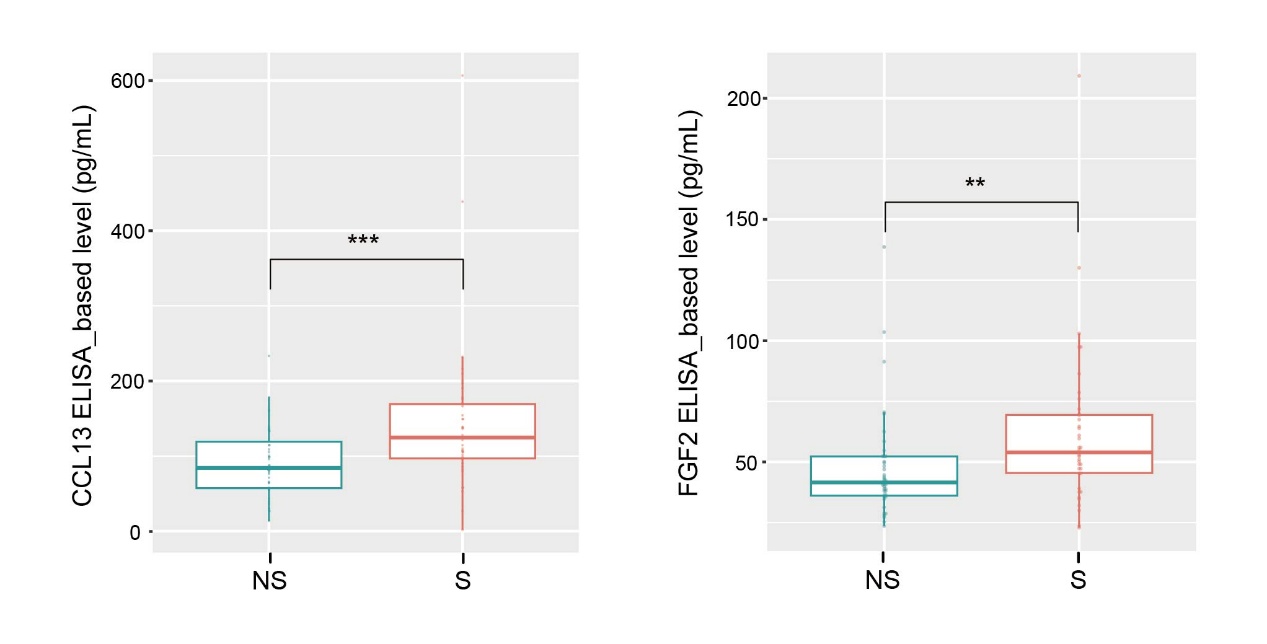


Fig. S7 Plasma protein levels of CCL13 and FGF2 measured by ELISA in the discovery cohort.


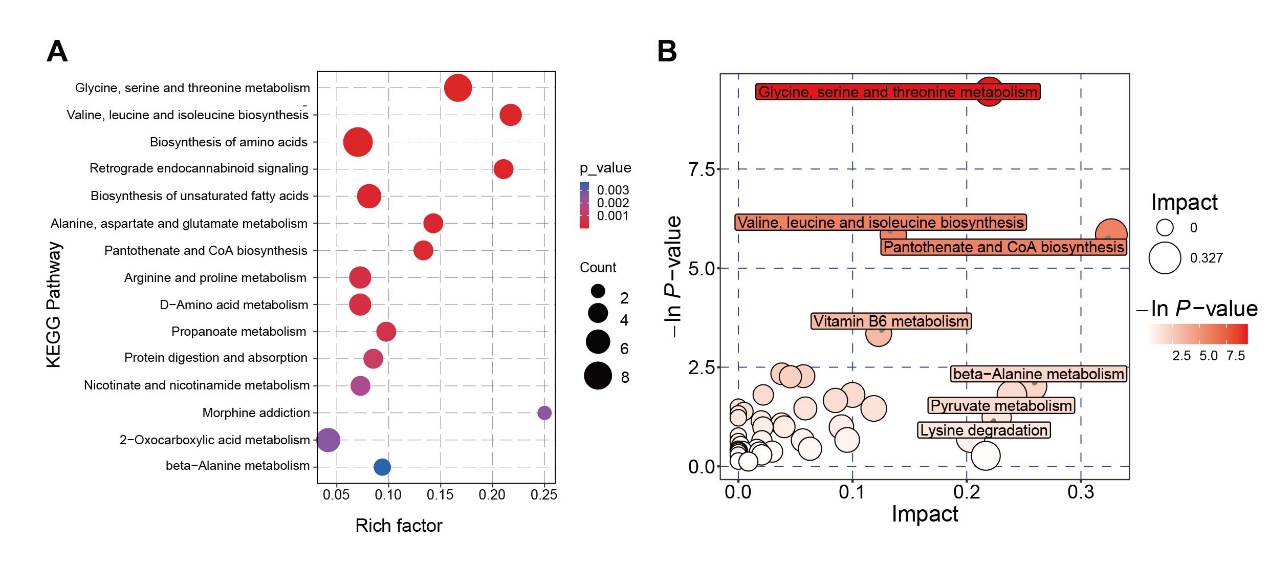


Fig. S8 (A) Bubble plots for KEGG pathways enrichment of differential metabolites. (B) Pathway topology analysis metabolites that distinguished sarcopenia from non- sarcopenia.


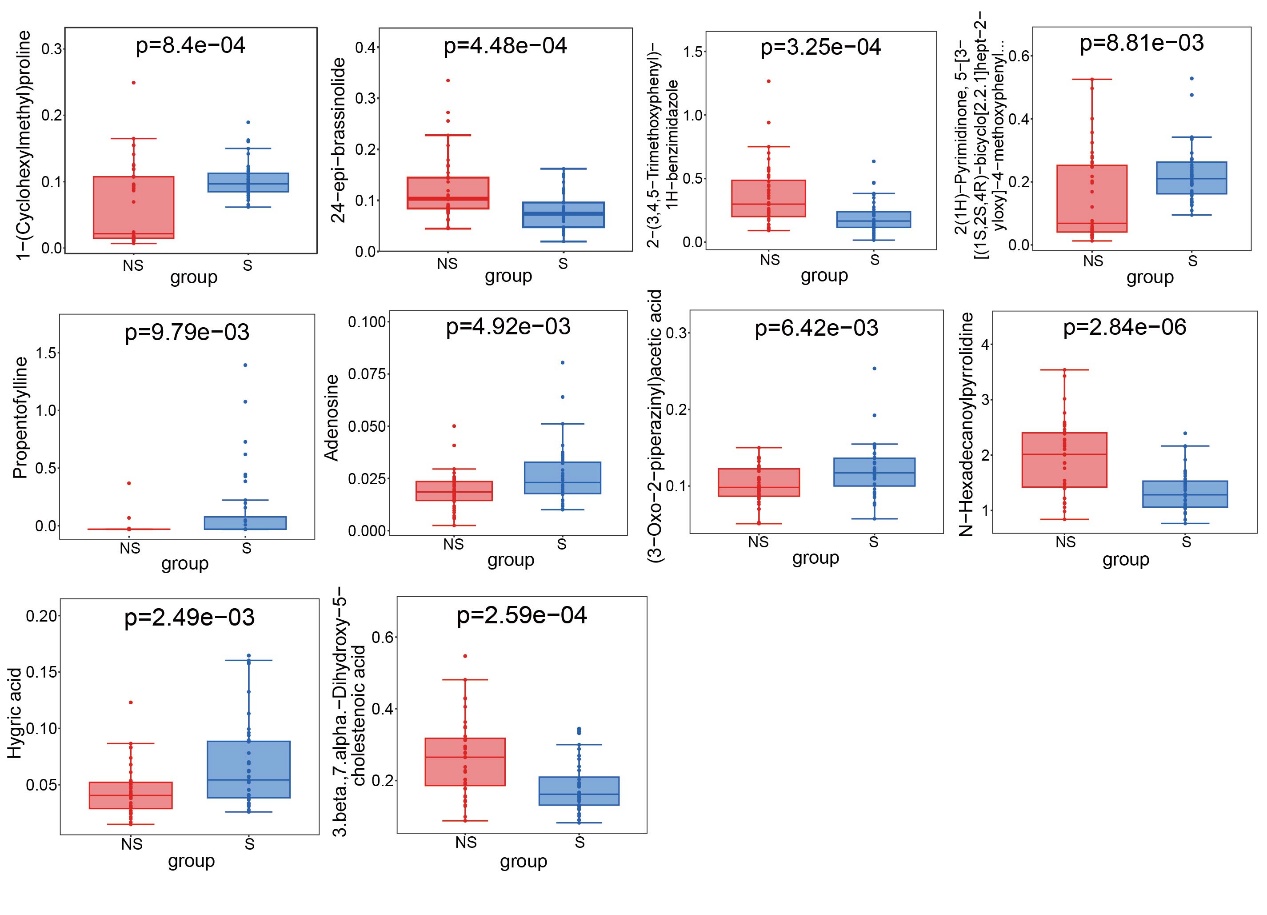


Fig. S9 Box plots showing the expression of the top 10 differential metabolites in S and NS in discovery cohort


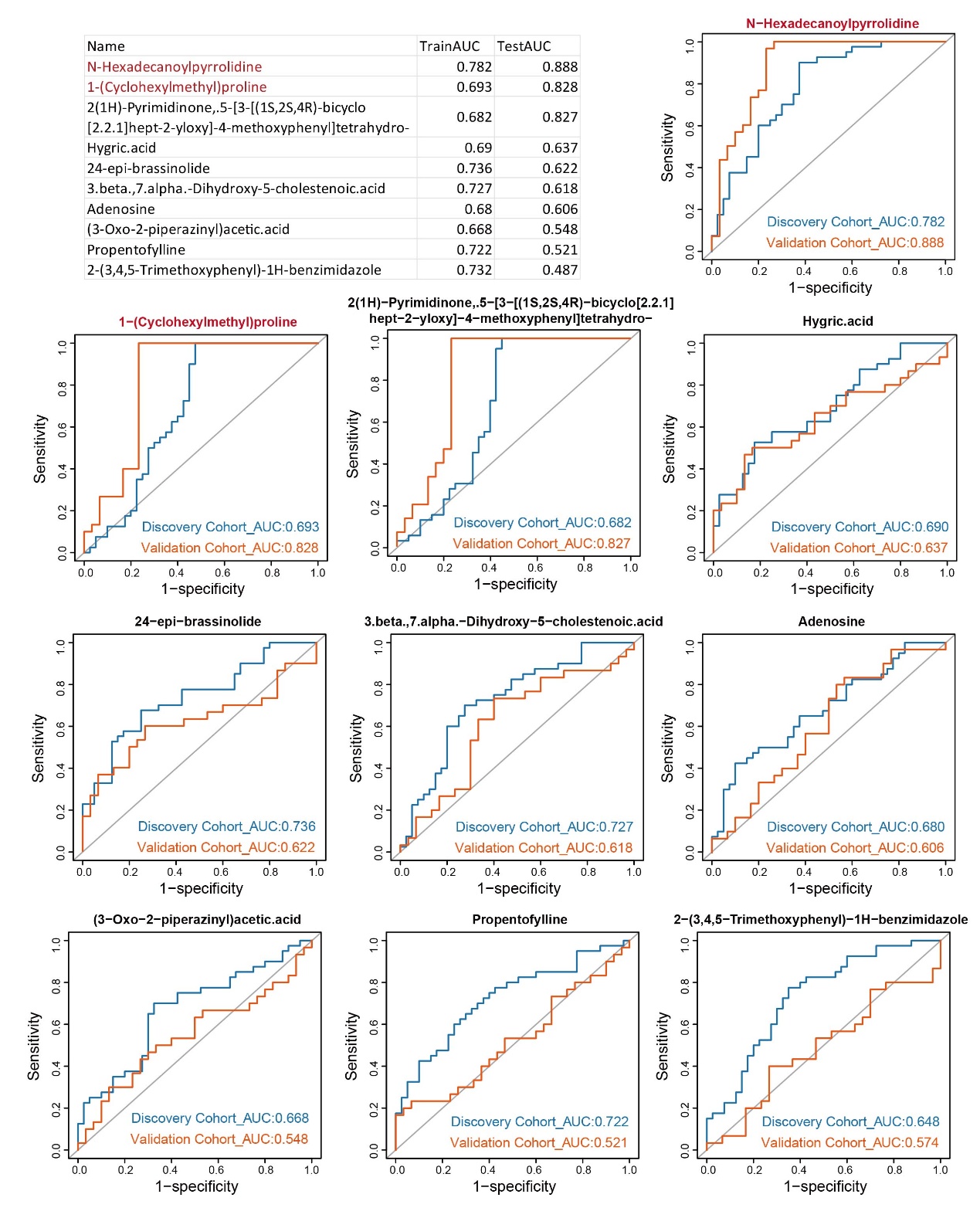


Fig. S10 ROC curve of the top 10 differential metabolites


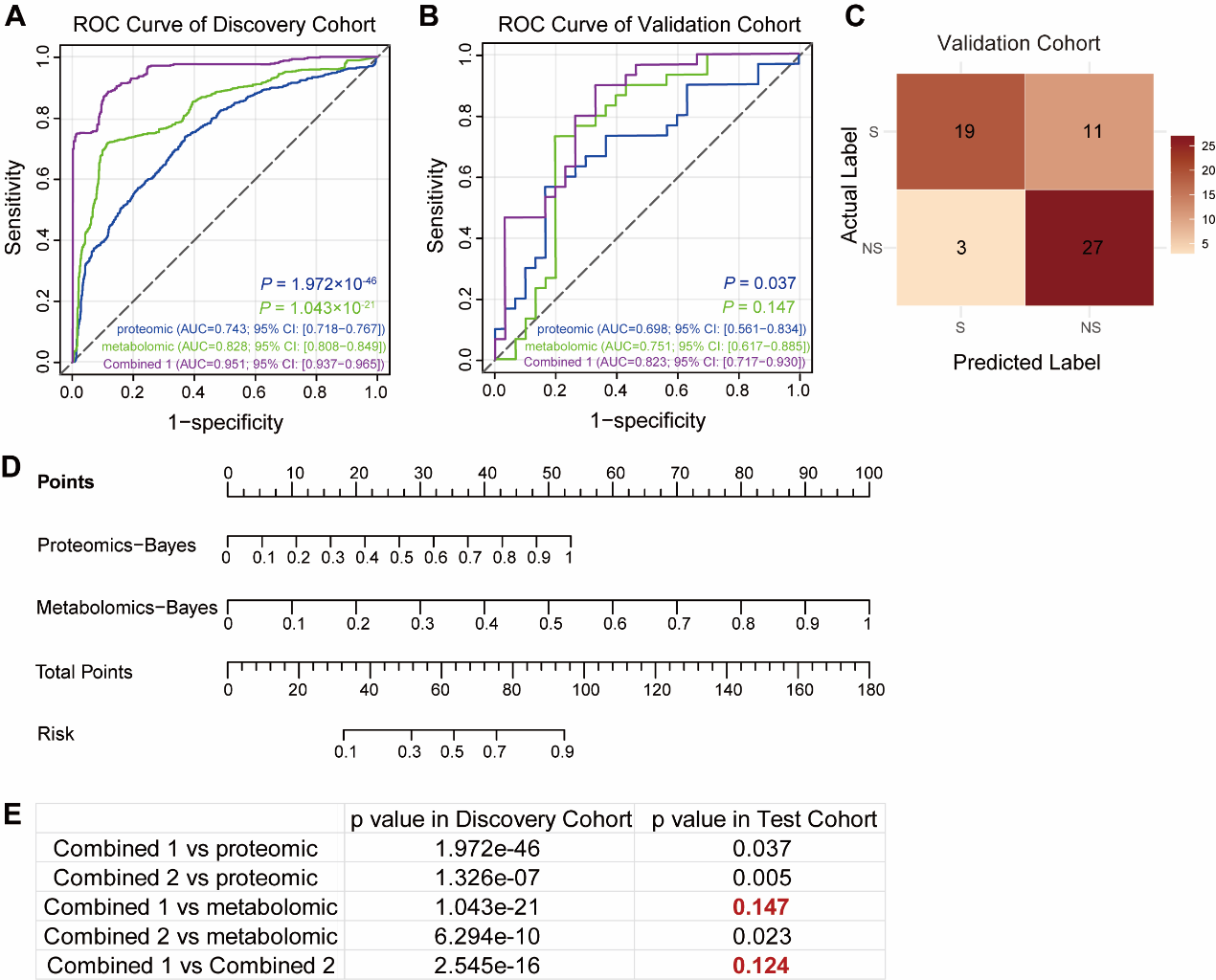


Fig. S11 Assessment of the prediction ability of proteomic, metabolomic models and Combined Model 1 for sarcopenia.
(A) ROC curves for proteomic, metabolomic models and Combined Model 1 in discovery cohort.

(B) ROC curves for proteomic, metabolomic models and Combined Model 1 in validation cohort.
(C) Confusion matrix analysis of Combined Model 1 in S versus NS group of validation cohort.
(D) Nomogram for the prediction of sarcopenia in whole population.
(E) Summary table of comparison values of AUC among groups.


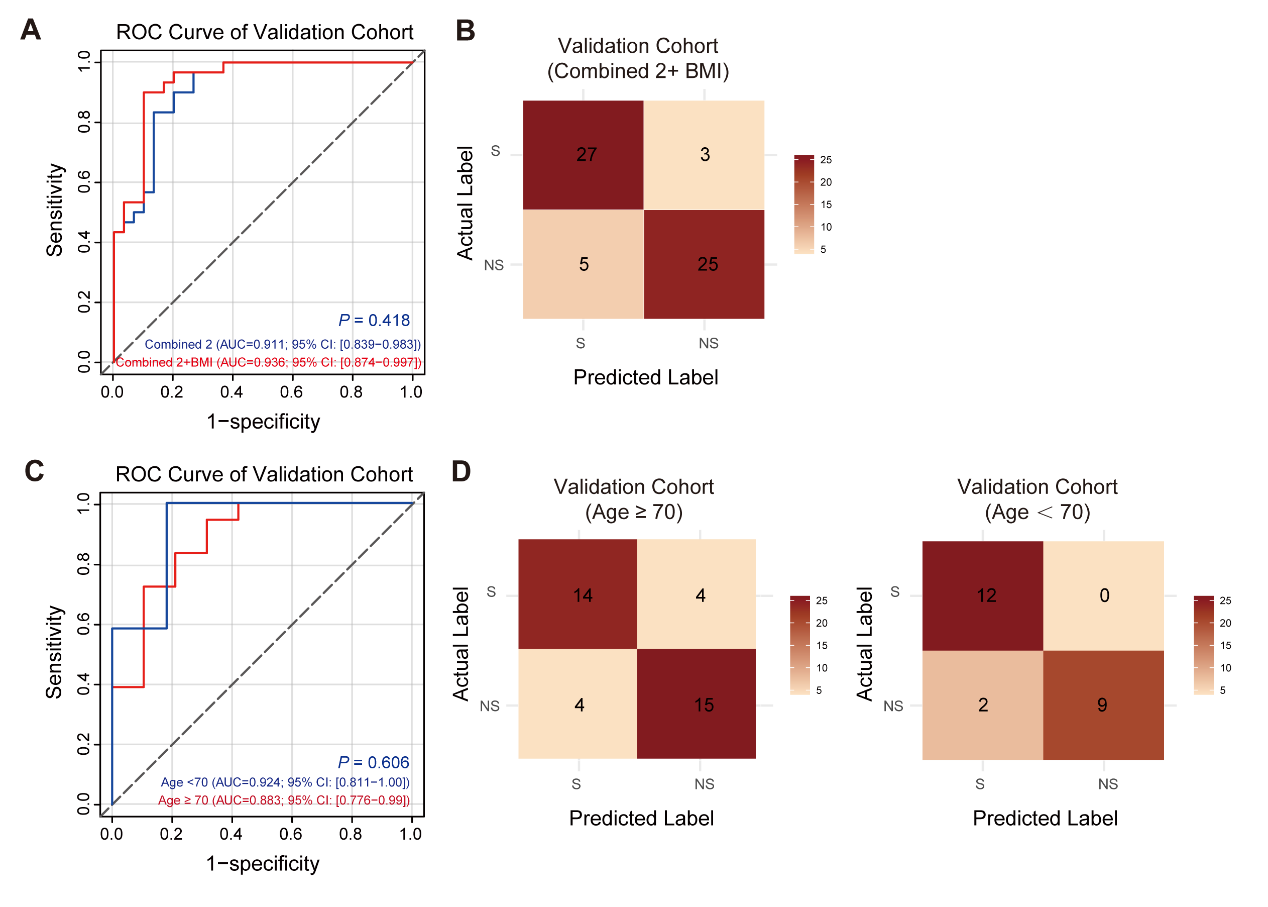


Fig. S12 Assessment of the model performance with BMI Augmentation and Age-Based Stratification.
(A) ROC curves for Combined 2 and Combined 2 + BMI models in validation cohort.

(B) Confusion matrix analysis of Combined 2 + BMI model in S versus NS group of validation cohort.

(C) ROC curves for the age-stratified Combined 2 model in the validation cohort.

(D) Confusion matrix analysis of Combined 2 (Age≥70) (left) and Combined 2 (Age＜70) (right) models in S versus NS group of validation cohort.

Table S1. Olink Explore 384 protein list.

Table S2. Differential expression results of proteins

Table S3. Performance of Six Machine Learning Proteomic Models

Table S4. Differential expression results of metabolomics data

Table S5. Diagnostic performance of Combined 2 + BMI, Combined 2 (Age≥70) and Combined 2 (Age＜70) models.

Table S6. Calibration Slope, Intercept, and Brier Score of proteomic, metabolomic models and Combined 2 Models.
